# Differential gene expression-based connectivity mapping identified novel drug candidate and improved Temozolomide efficacy for Glioblastoma

**DOI:** 10.1186/s13046-021-02135-x

**Published:** 2021-10-25

**Authors:** Raghupathy Vengoji, Pranita Atri, Muzafar A. Macha, Parthasarathy Seshacharyulu, Naveenkumar Perumal, Kavita Mallya, Yutong Liu, Lynette M. Smith, Satyanarayana Rachagani, Sidharth Mahapatra, Moorthy P. Ponnusamy, Maneesh Jain, Surinder K. Batra, Nicole Shonka

**Affiliations:** 1 Department of Biochemistry and Molecular Biology, University of Nebraska Medical Center, Omaha, NE 68198-5870 USA; 2 Watson-Crick Centre for Molecular Medicine, Islamic University of Science and Technology, Jammu & Kashmir, India; 3 Department of Radiology, University of Nebraska Medical Center, Omaha, NE 68198-5870 USA; 4 Department of Biostatistics, University of Nebraska Medical Center, Omaha, NE 68198-5870 USA; 5 Fred and Pamela Buffett Cancer Center, University of Nebraska Medical Center, Omaha, NE 68198-5870 USA; 6 Department of Pediatrics, University of Nebraska Medical Center, Omaha, NE 68198-5870 USA; 7 Eppley Institute for Research in Cancer and Allied Diseases, University of Nebraska Medical Center, Omaha, NE 68198-5870 USA; 8 Department of Internal Medicine, Division of Oncology & Hematology, University of Nebraska Medical Center, Omaha, NE 68198 USA

**Keywords:** Connectivity map, In silico analysis, Glioblastoma, HDAC, Blood-brain barrier

## Abstract

**Background:**

Glioblastoma (GBM) has a devastating median survival of only one year. Treatment includes resection, radiation therapy, and temozolomide (TMZ); however, the latter increased median survival by only 2.5 months in the pivotal study. A desperate need remains to find an effective treatment.

**Methods:**

We used the Connectivity Map (CMap) bioinformatic tool to identify candidates for repurposing based on GBM’s specific genetic profile. CMap identified histone deacetylase (HDAC) inhibitors as top candidates. In addition, Gene Expression Profiling Interactive Analysis (GEPIA) identified HDAC1 and HDAC2 as the most upregulated and HDAC11 as the most downregulated HDACs. We selected PCI-24781/abexinostat due to its specificity against HDAC1 and HDAC2, but not HDAC11, and blood-brain barrier permeability.

**Results:**

We tested PCI-24781 using in vitro human and mouse GBM syngeneic cell lines, an in vivo murine orthograft, and a genetically engineered mouse model for GBM (*PEPG* - PTEN^flox/+^; EGFRvIII+; p16^Flox/−^ & GFAP Cre +). PCI-24781 significantly inhibited tumor growth and downregulated DNA repair machinery (BRCA1, CHK1, RAD51, and O^6^-methylguanine-DNA- methyltransferase (MGMT)), increasing DNA double-strand breaks and causing apoptosis in the GBM cell lines, including an MGMT expressing cell line in vitro. Further, PCI-24781 decreased tumor burden in a PEPG GBM mouse model. Notably, TMZ + PCI increased survival in orthotopic murine models compared to TMZ + vorinostat, a pan-HDAC inhibitor that proved unsuccessful in clinical trials.

**Conclusion:**

PCI-24781 is a novel GBM-signature specific HDAC inhibitor that works synergistically with TMZ to enhance TMZ efficacy and improve GBM survival. These promising MGMT-agnostic results warrant clinical evaluation.

**Supplementary Information:**

The online version contains supplementary material available at 10.1186/s13046-021-02135-x.

## Topic

Connectivity Map, In silico analysis, Glioblastoma, GBM mice, syngeneic cell line.

## Key points

Identifying drugs via connectivity mapping and evaluation of these novel drugs can substantially reduce the time to drug discovery.

PCI-24781 enhanced the efficacy of TMZ in GBM by targeting DNA repair machinery.

## Background

Glioblastoma (GBM) is the most common primary brain malignancy in adults [1]. Current treatments have not significantly improved the overall survival (OS) of GBM patients in the past decade. Adding temozolomide (TMZ) to radiation therapy (RT), only increased median survival by 2.5 months overall [2], and by 6.4 months in cases exhibiting epigenetic silencing of DNA repair enzyme O^6^-methylguanine-DNA-methyltransferase (MGMT) [3]. TMZ and radiation-induced DNA damage are repaired by the DNA repair pathway, upregulated in GBM [4]. Importantly, TMZ resistance is observed even in the absence of MGMT, as Base Excision Repair (BER), mismatch repair (MMR), and p53 mutations heavily influence TMZ sensitivity [5]. Thus, a drug targeting the specific GBM signature agnostic of MGMT or DNA repair enzyme status could lead to synergistic cytotoxicity with TMZ.

Histone acetylation is regulated by histone acetyltransferase (HAT) and histone deacetylase (HDAC), resulting in chromatin structure alterations and increased accessibility to DNA for transcriptional activation or repression [6, 7]. Hyperacetylation of heat shock protein 90 (HSP90) alters its chaperone function and facilitates polyubiquitination and proteasomal degradation of HSP90 target proteins [8]. Pan-HDAC inhibitors panobinostat and vorinostat/suberoylanilide hydroxamic acid (SAHA) cause hyperacetylation of nuclear HSP90, degrading DNA repair machinery proteins BRCA1, ATR, and CHK1 in breast cancer cells [9]. PCI-24781 increased the radiosensitization of pediatric GBM cells by decreasing the DNA repair machinery proteins RAD51, Ku70, Ku86 and DNA-PKcs [10]. In addition, HDAC inhibitors MS275 and TSA increased the sensitivity of TMZ and lomustine (CCNU) respectively, by inducing apoptosis in GBM cells [11, 12]. Interestingly, the HDAC inhibitor RGFP109 increased the sensitivity of TMZ even in TMZ resistant GBM cell lines by inhibiting the NF-kB pathway [13]. HDAC inhibitors significantly reduced GBM growth in preclinical studies [14]. Romidepsin/FK228 significantly reduced the tumor growth possibly by inhibiting PI3K/AKT/mTOR pathways [15]. Disappointingly, in clinical trials, these drugs did not improve OS [16, 17] due to: (a) lack of inhibitor selectivity, (b) poor blood-brain barrier (BBB) permeability, (c) limited efficacy, and (d) high toxicity [18, 19]. We acknowledge that phase I clinical trials are designed to demonstrate safety rather than efficacy and that testing in recurrent tumors likely to be resistant to TMZ may not be optimal for observing efficacy, however the toxicity and overall results of these studies did not support further testing in GBM.

We used an in silico approach using Connectivity Map (CMap), a tool developed by the BROAD Institute, to identify drugs for repurposing based on their effect on tumors’ genetic profiles [20]. We identified the BBB-permeable PCI-24781/abexinostat/THM-I-94, which reverses the GBM gene signature by inhibiting HDAC1 and 2, while not affecting HDAC11. We evaluated its anticancer activities using human and mouse GBM cell lines in vitro and mouse orthograft and transgenic (*PEPG* - PTEN^flox/+^; EGFRvIII+; p16^Flox/−^ & GFAP Cre +)  in vivo models. PCI-24781 enhanced apoptosis and downregulated DNA repair machinery (RAD51, CHK1, and MGMT) in GBM cell lines in vitro. Further, PCI-24781 with TMZ decreased tumor burden and increased OS in orthotopic murine models compared to vorinostat plus TMZ. Importantly, PCI-24781 also decreased the tumor volume in a 4.5-month-old genetically engineered mouse (*PEPG* - PTEN^flox/+^; EGFRvIII+; p16^Flox/−^ & GFAP Cre +). Our approach identified a novel, selective HDAC inhibitor capable of potentiating the effects of TMZ in GBM tumors, agnostic of DNA repair enzyme status.

## Methods

### In silico analysis

#### Identification of GBM datasets

Searching the NCBI repository Gene Expression Omnibus (GEO) datasets using the keywords “GBM” with “*Homo sapiens*” and “tissue” identified 6183 studies. Four datasets were selected for treatment-naïve tumors containing both normal (N) and tumor (T) samples within the same dataset. GSE61335 (T- 48; N - 14), GSE35493 (T - 12; N - 8), GSE50161 (T - 34; N – 13) and GSE13276 (T – 5; N – 3) [21–24]. The datasets with mixed samples were filtered to identify GBM specific samples. We considered 99 tumors and 38 normal or adjacent brain samples. To identify the differential gene expression in T vs. N, a linear model (Limma package -R- Bioconductor) was used for each of the studies separately [25].

#### Identification of potential therapeutics using CMap

Limma identified differentially expressed genes between normal and tumor samples. The top 150 upregulated and top 150 downregulated genes from each dataset were subjected to a CMap query to identify negatively connected drugs able to reverse the GBM signature. Comparing the negatively connected drugs identified using each dataset led to 12 common drugs from which PCI-24781 was chosen based on its selectivity, apoptotic potential, and ability to cross the BBB.

#### In-silico identification of pathways affected by high scoring HDAC inhibitors

The signatures of the high scoring HDAC inhibitors (PCI-24781, vorinostat, belinostat) were assessed using the web-server iLINCS [26]. Further, the web-based search tool for interactions of chemicals (STITCH) was used to assess chemical and protein interactions [27]. The top pathways from each drug network were analyzed further.

#### GBM cell lines and cell cultures

GBM cell line cultures are described in Supplementary Methods. Cell line validation was done at the University of Arizona genetics core, Tucson, AZ. USA, by PCR-based short-tandem repeat (STR) analysis.

#### Inhibitor

PCI-24781 was obtained from Xynomic Pharmaceuticals, USA, was dissolved in DMSO and aliquots stored in − 20^•^C.

#### Combination index

The combination index/effect (CI) of TMZ and PCI-24781 was determined using CompuSyn software [28]. A CI value less than 1 supports synergy whereas > 1 confers antagonist interaction.

#### Generation of the U-118MG brain tumor xenografts

All animal experiments were reviewed and approved by the Institutional Animal Care and Use Committee. U-118MG tumor xenografts were generated as described with slight modifications [29] (see Supplementary Methods).

### GBM mouse model development

A transgenic mouse expressing Cre recombinase under the control of the astrocyte-specific human GFAP promoter (B6.Cg-Tg(GFAP-cre) 8Gtm/Nci (B6); strain number 01XN3), EGFRvIII mouse (CAG-LSL-EGFRvIII; strain number 01X68) were obtained from the NCI mouse repository. PTEN mouse (B6.129S4-Ptentm1Hwu/J; strain number 006440) was obtained from Jackson Laboratory and p16 mouse (EM:00435 FVB;129P2-Cdkn2atm1.1Brn/Cnrm; strain number EM:00435) obtained from the European Mouse Mutant Archive (EMMA).

Animals were maintained with food and water ad libitum and kept in 12 h alternating dark/light cycle. All animal experiments were carried out in accordance with U.S. Public Health Service, “Guidelines for the care and use of laboratory animals”, in accordance to ARRIVE guidelines and with approval of the UNMC Institutional Animal Care and Use Committee (IACUC) (UNMC IACUC # 20–028-04 FC). We genotyped mice for EGFRvIII and Cre positivity, p16 (point mutation in exon 2 of p16Ink4a) and PTEN exon 5 deletions by PCR. Genotyping primers and PCR conditions were followed as described by the animal suppliers. Tumor growth was measured by MRI bimonthly. Mice were sacrificed when they lost 20% body weight or paralysis occurred and their brains were fixed with 10% neutral-buffered formalin; brain tissues were processed into paraffin blocks for hematoxylin, eosin, and immunohistochemistry staining for lineage markers (glial and neural). Our pathologist confirmed that these tumors (*PEPG* - PTEN^flox/+^; EGFRvIII+; p16^Flox/−^ & GFAP Cre +) were high-grade gliomas/GBM.

#### Syngeneic cell line development

Mice were sacrificed at ages 4 and 6 months (when mice were weak/paralyzed), and a part of tumor tissue was utilized for tumor characterization and RNA and protein isolation, and the rest of the tumor was used for cell line generation as described earlier [30]. Cell line characterization (PTEN & p16- Genotyping PCR; EGFRvIII – Immunoblotting with EGFRvIII specific antibody; p53 mutation – Sequencing), tumorigenic potential in vitro and in vivo were done after 15 passages in cell culture.

#### Magnetic resonance imaging (MRI) and data analysis

MRI was performed on a 7 Tesla scanner (Bruker PharmaScan, Billerica, MA) operated by ParaVision 7 with a quadrature RF coil for signal transmission and reception. T2-weighted images were acquired at the axial direction using a TuborRARE sequence with the following parameters: TR/TE = 4200/48 ms, RARE factor = 8, Averaging = 4, Matrix size = 256 × 192, FOV = 20 × 20 mm^2^, slice number = 21, slice thickness = 0.5 mm. T1-weighted MRI was performed using a MDEFT sequence with 4 segments, segment TR = 2600 ms, TE = 2.2 ms, TI = 950 ms, flip angle = 30°, Averaging = 3, Matrix = 256 × 256, FOV = 20 × 20 mm^2^, slice number = 15, slice thickness = 0.5 mm. The T1- and T2-weighted MRI was followed by tail vein injection of gadolinium (MultiHance, Bracco Diagnostics) at 0.1 mmol/kg. After 15 min of gadolinium injection, the mouse was scanned using T1- and T2-weighted MRI again. Mice were anesthetized using 1.5% isoflurane carried by 1 L/min oxygen. The breathing rate and body temperature were monitored during scanning. The isoflurane was continuously adjusted to maintain the breathing rate between 40 and 80 bpm.

Region-of-interest (ROI) analysis in image J (imagej.nih.gov/ij/) was used to measure tumor volumes. The post-contrast T1-weighted images (T1post) were primarily used for the measurements with pre-contrast T1-weighted (T1pre) and T2-weighted images as references.

### Statistical analysis

The in vitro assays were repeated at least three times and are presented as mean values ± SD. ANOVA was used to evaluate differences between groups, and Tukey’s method was used to adjust for multiple comparisons. Photon data on Day 32 were analyzed on the natural log scale to meet model assumptions. Pairwise comparisons between control and treatment groups were adjusted for multiple comparisons with Dunnett’s photon data method. Overall survival was estimated using the Kaplan-Meier method, and groups were compared using the log-rank test. Mice alive at the end of the study were treated as censored. *P*-values less than 0.05 were considered as statistically significant. Statistical analyses were performed using SAS 9.4 (SAS Institute Inc., Cary, NC).

## Results

### In silico connectivity mapping (CMap) identified potential drugs for GBM

Initial analysis of GEO datasets identified 18,457 human GBM studies, of which 6183 were tissue-based and further restricted for 1) treatment-naïve tumor samples (to preserve native tumor gene expression profile) and 2) common dataset expression profile of normal (N) and tumor (T) (to fit a linear model). Four datasets were selected: GSE61335 (T- 48; N - 14), GSE35493 (T - 12; N - 8), GSE50161 (T - 34; N - 13) and GSE13276 (T - 5; N - 3) for 99 tumors and 38 normal or adjacent normal brain samples.

**Fig. 1:**
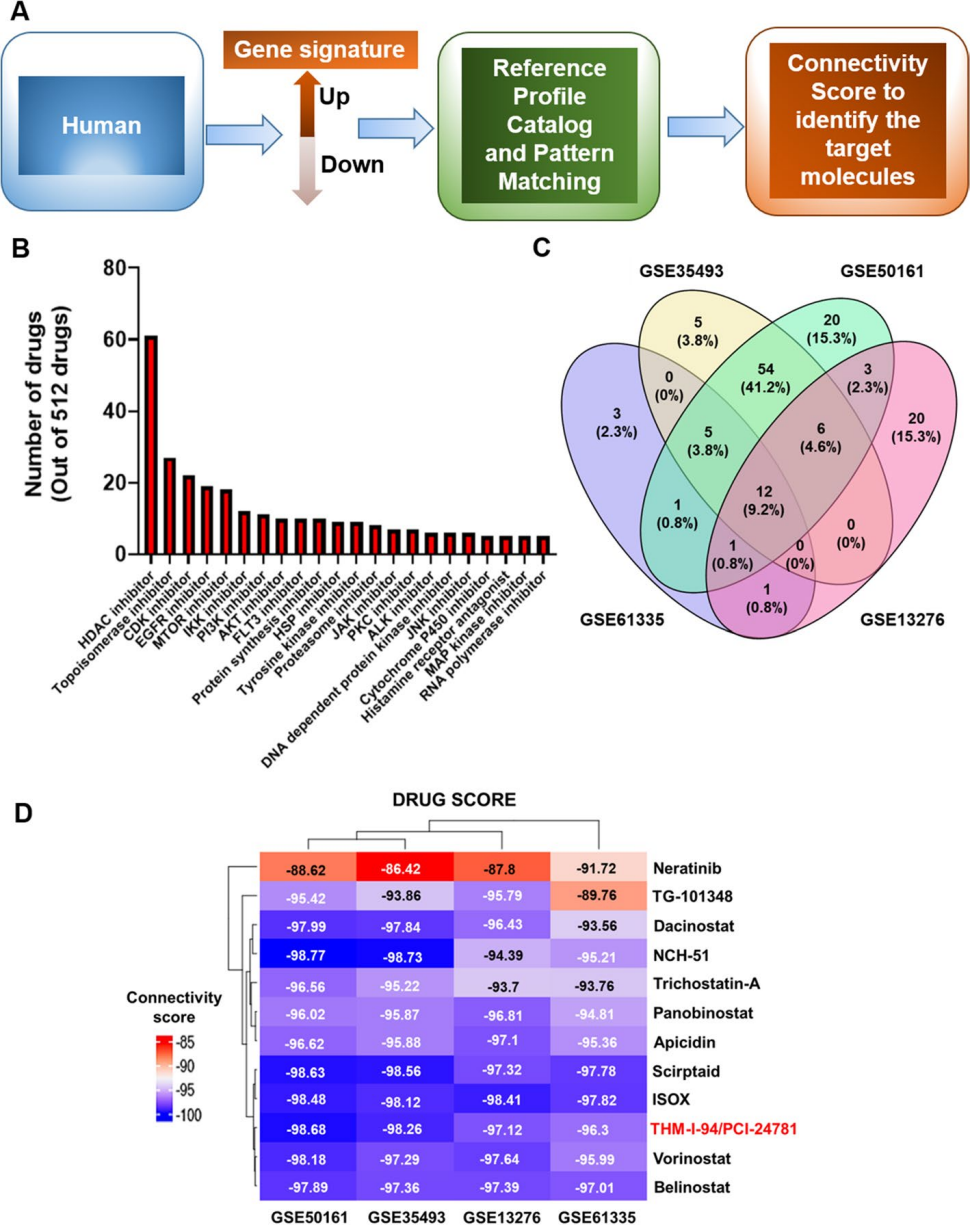
Drug classes negatively connected to the GBM signature. **A** Pipeline for drug discovery using connectivity mapping. **B** Drug classes with negative connectivity scores (> − 80). HDAC inhibitors were found to be the most enriched class. **C** The negatively connected drugs from CMap compared with a four-way Venn diagram. The 12 drugs negatively connected to all datasets were then studied more closely. **D** Top 12 drugs exhibiting negative connection with GBM signature common across the four datasets

**Fig. 2:**
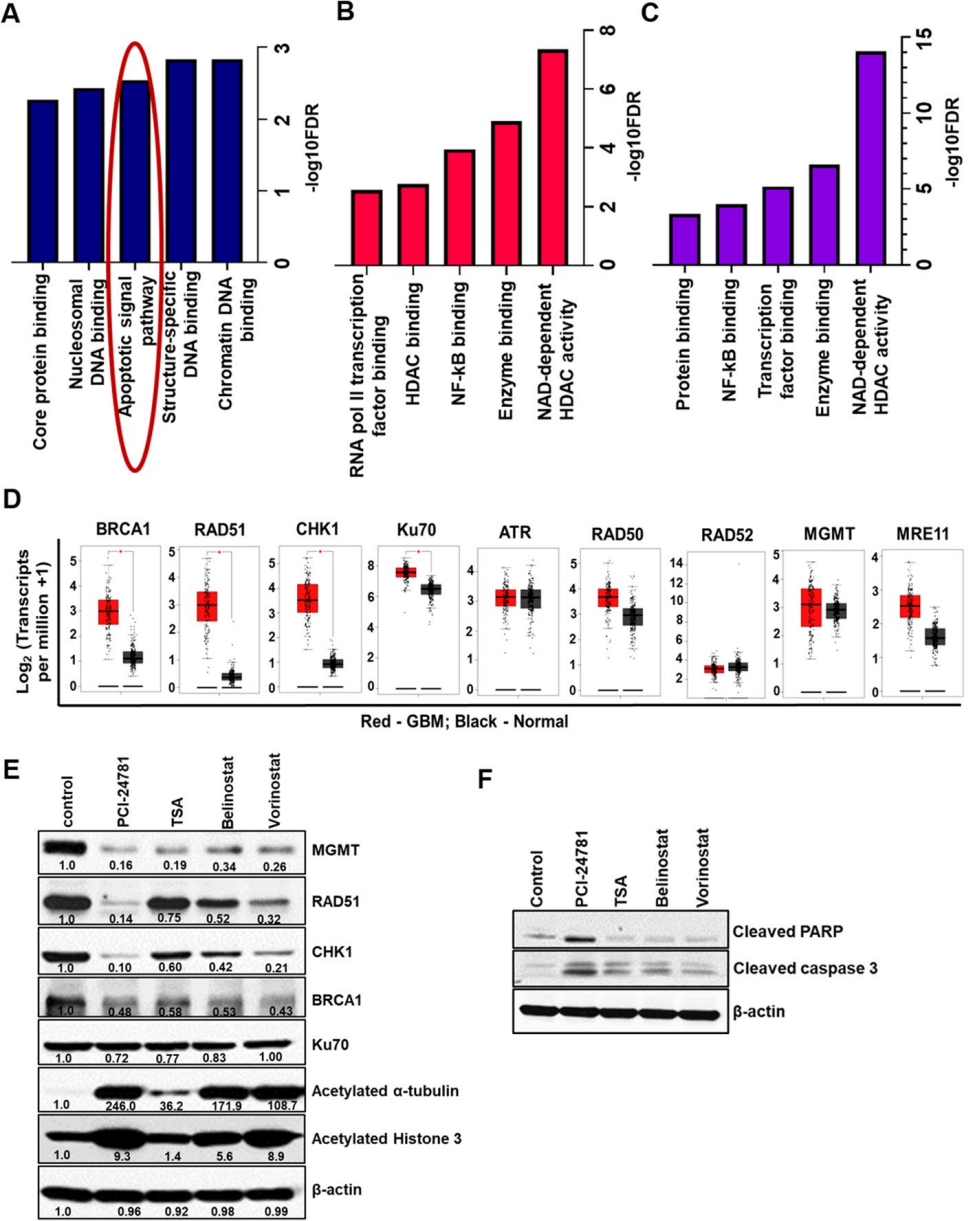
PCI-24781 potentially decreases the DNA repair machinery and activates apoptotic signaling (**A-C**) Pathways: The web-based iLINCS server was used to assess the associated signatures for each of the top 3 scoring drugs. Pathways affected by PCI-24781 (**A**), belinostat (**B**), and vorinostat (**C**). Interestingly, PCI-24781 has a unique signature when compared to pan-HDAC inhibitors and specifically impacts apoptotic signaling. **D** DNA repair machinery expression in GBM Using GEPIA on 163 GBM tumors (red) and 207 controls (black) show that DNA repair machinery genes BRCA1, RAD51, CHK1, and Ku70 are significantly upregulated in GBM when compared to control (*p* < 0.01). **E** PCI-24781 effectively decreases the DNA repair machinery in U-118MG GBM cells. U-118MG cells were treated with 1.5 μM of HDAC inhibitor for 48 h, and lysates were analyzed for DNA repair machinery proteins by immunoblotting. The extent of acetylation of α tubulin supports the efficacy of PCI-24781. DMSO served as vehicle control. **F** U-118MG cells were treated with 1.5 μM of HDAC inhibitor for 48 h, and lysates were analyzed for apoptotic markers cleaved PARP (*CST- 9541*; recognize cleaved PARP, not total) and cleaved caspase 3

To identify differential gene expression in T vs. N, a linear model (Limma package) was used for each study [25]. Those genes were matched to gene expression profiles from over 2300 drugs in CMap Fig. 1A. Five-hundred twelve drugs were highly negatively connected to each dataset (Fig. 1B), most of which were HDAC inhibitors. We then assessed CMap scores for connections to gene signatures for all twelve drugs common to the four datasets from the updated CMap data (Fig. 1C, D & Table S1). The highest scoring cluster (Fig. 1D) included PCI-24781, vorinostat, and belinostat. A drug-protein network using the web-server iLINCS and STITCH identified specific pathways affected by each drug (Fig. 2A - C). Unlike vorinostat and belinostat, the PCI-associated gene network showed significant differences in DNA binding and apoptotic signaling, corroborated by western blot analysis (Fig. 2A – C & F).

Further, Gene Expression Profiling Interactive Analysis (GEPIA) [31] comparing normal (207) and GBM (163) samples revealed significant upregulation of DNA repair machinery genes BRCA1, CHK1, RAD51, and Ku70 in GBM (*p* < 0.01) (Fig. 2D). In MGMT expressing U-118MG cells, PCI-24781 potentially downregulated DNA repair machinery proteins MGMT, RAD51, and CHK1 compared to pan-HDAC inhibitors, suggesting that relative TMZ resistance (Fig. 2E) -possibly due to a higher expression of these DNA repair enzymes - may be overcome by PCI-24781.

**Fig. 3:**
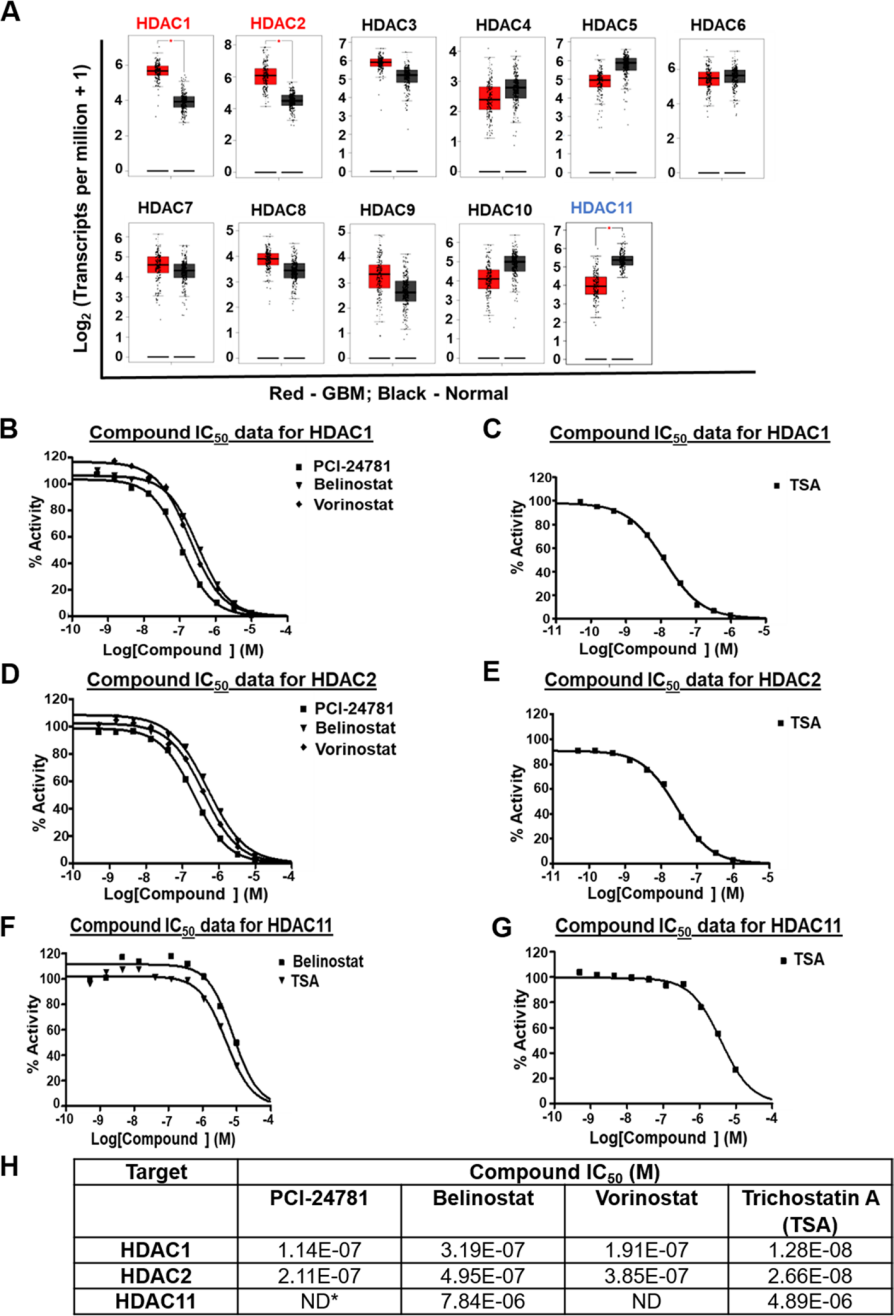
PCI-24781 does not inhibit HDAC11. **A** GEPIA on HDAC expression in GBM (Sample size; Control (Black) (*n* = 207) and GBM (Red) (*n* = 163)). HDAC1 and 2 are significantly upregulated, while HDAC11 is downregulated (p < 0.01). GEPIA- Gene Expression Profiling Interactive Analysis. **B–H** HDAC1, HDAC2, and HDAC11 assay were done by Reaction Biology Corp. using fluorogenic peptide from p53 residues 379–382 (RHKK(Ac)AMC) [substrates for HDAC1 and 2] and trifluoroacetyl lysine [substrate for HDAC11]. PCI-24781 and vorinostat inhibit HDAC1 and 2 but do not have an inhibitory effect on HDAC11, as do belinostat and TSA. **B, D** & **F** HDAC reference compound TSA were tested in a 10 dose IC_50_ with 3 fold serial dilution starting at 10 μM (**C**) HDAC1, (**E**) HDAC2 and (**G**) HDAC11. (**H**) IC_50_ values are summarized in table.*Empty cells indicate no inhibition or compound activity that could not fit an IC_50_ curve. TSA-Trichostatin

GEPIA also revealed a specific GBM HDAC signature: significant upregulation of HDAC1 and HDAC2 with concomitant downregulation of HDAC 11 (p < 0.01) (Fig. 3A). PCI and vorinostat were found to concurrently inhibit HDAC 1 and 2, but not HDAC11 (Fig. 3B- H, Table S2). As PCI-24781 had never been evaluated in adult GBM, exhibits inhibitor selectivity, and effectively downregulates DNA repair enzymes, we elected to further study and characterize its effects.

### PCI-24781 inhibits GBM cell proliferation in vitro

We evaluated the efficacy of PCI-24781 on GBM cell proliferation with different genetic backgrounds: U87 (p53 WT and MGMT methylated), U251 (p53 mutated and MGMT methylated), and U-118MG (p53 mutated and MGMT un-methylated) using an MTT assay. The inhibitory concentration (IC_25_) values (0.5–1.25 μM) were similar among these cell lines, and its anti-tumorigenic potential was not altered by p53 mutational status (Table S3). Enticingly, PCI-24781 was equally effective in MGMT-expressing U-118MG cells and relatively chemoresistant EGFRvIII-expressing cells (Fig. S1A & B; Table S3) [32]. Further, PCI-24781 decreased cell proliferation of our mouse syngeneic cell line (EGFRvIII+; p16^Flox/Flox^ & GFAP Cre +), resistant to TMZ, in a dose-dependent manner (Fig. S1C & D).

### PCI-24781 decreases in vitro tumorigenicity of GBM cells

**Fig. 4:**
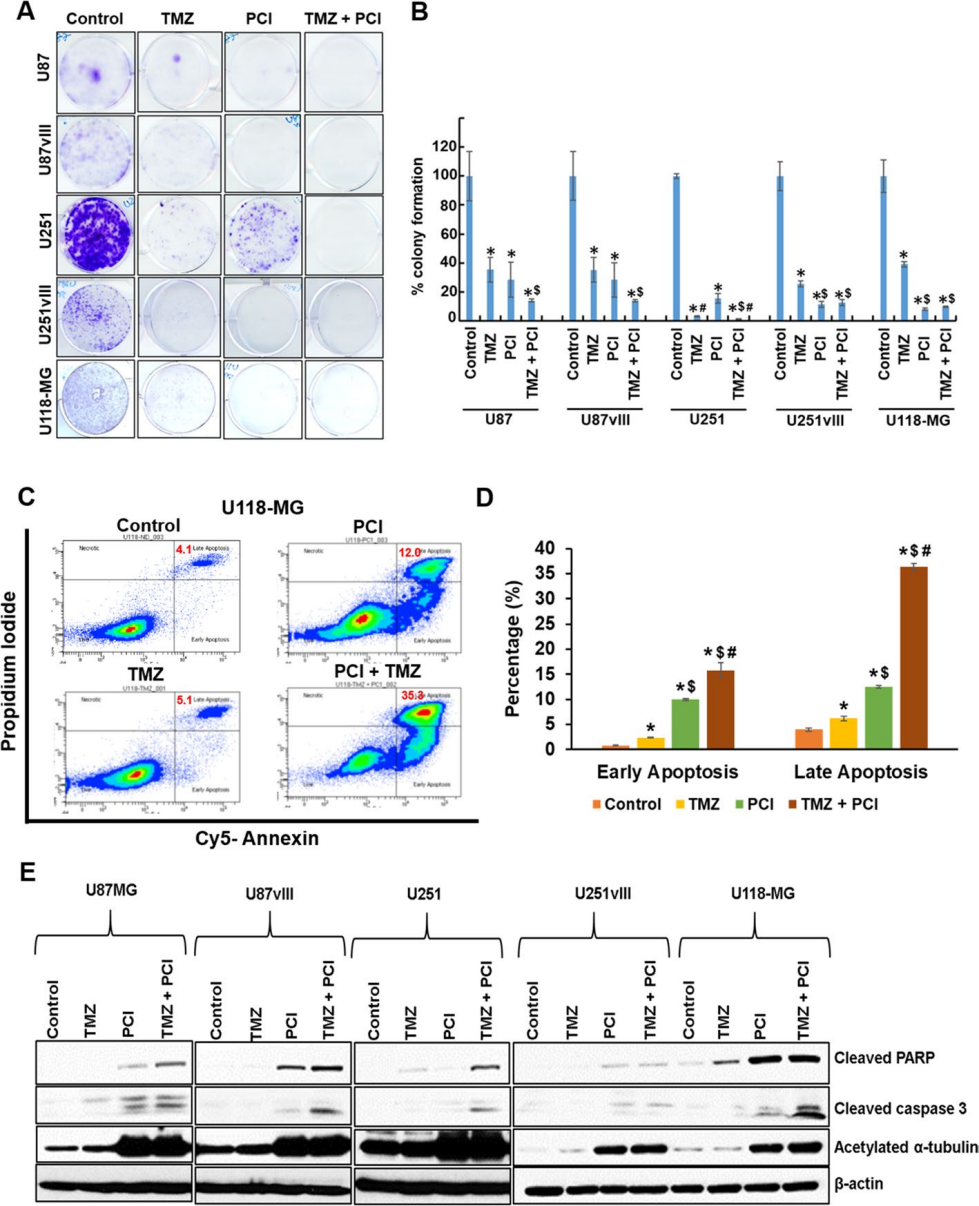
PCI-24781 suppresses GBM cell proliferation and induces apoptosis. Cells were seeded in DMEM complete media. After 12 h, cells were treated with vehicle control, IC_25_ of TMZ, IC_25_ of PCI-24781, or TMZ and PCI-24781 for 48 h, and cultured up to 2 weeks in drug-free DMEM complete media. Colonies were fixed with methanol and stained with crystal violet, then dissolved in 10% acetic acid, and absorbance measured at 595 nm. Results represented as difference in percentage colony formation. **A** Representative images of colony formation assay. **B** Mean percent difference in colony formation in different drug treatment groups from three independent experiments. ANOVA was used to compare the colony formation variable on the natural log scale. Pairwise comparisons are adjusted with Tukey’s method. ‘*’ *p* ≤ 0.0001; ‘$’ *p* ≤ 0.01; ‘#’ *p* < 0.001 “*” significantly different compared to vehicle control; “$” significantly different compared to TMZ; “#” significantly different compared to PCI-24781. **C**-**E** PCI-24781 induces apoptosis in GBM cells. **C** Representative Annexin V and PI staining of vehicle control, TMZ, PCI, and TMZ + PCI treated U-118MG cells. **D** Quantification (percentage of) early and late apoptotic cells. Early and late apoptosis was compared between groups using ANOVA. Pairwise comparisons are adjusted with Tukey’s method. ‘*’ *p* < 0.03; ‘$’ *p* < 0.0001; ‘#’ *p* < 0.0001. “*” significantly different compared to vehicle control; “$” significantly different compared to TMZ; “#” significantly different compared to PCI-24781. **E** U87, U87EGFRvIII, U251, U251EGFRvIII, and U-118MG cells were incubated with the drugs mentioned above for 48 h, and lysates were subjected to immunoblot analysis to identify the levels of apoptotic proteins, cleaved PARP, and cleaved caspase-3

We analyzed PCI-24781 on GBM cell in vitro tumorigenicity using colony formation assay. Treatment of U87, U87EGFRvIII, U251, U251EGFRvIII, and U-118MG cells with PCI-24781 resulted in significant suppression of colony formation compared to vehicle control (*p* < 0.001) (Fig. 4A & B) and resulted in 71 ± 12; 72 ± 12; 84 ± 3; 89 ± 2; 92 ± 1 percentage decrease in colony formation in these cell lines respectively. Though some colonies were resistant to monotherapy, combination treatment completely abrogated GBM colony formation. PCI-24781 significantly decreased the in vitro tumorigenic capability of U-118MG cells compared to TMZ (*p* < 0.0001) (Fig. 4A & B). These results support the anti-tumorigenic potential of PCI-24781 on MGMT expressing GBM cells. Further, combining PCI-24781 + TMZ significantly decreased the tumorigenic potential of EGFRvIII+; p16^Flox/Flox^ & GFAP Cre + syngeneic cell lines (Fig. S2A & B).

### Effect of PCI-24781 on GBM cell viability

We next investigated the correlation between PCI’s demonstrated ability to suppress tumor growth and apoptosis. Our PI/Annexin V apoptosis assay on U-118MG cells revealed that single-agent PCI-24781 induced significant cell death (p < 0.0001). The percentages of early and late apoptotic cells together were (4.8 ± 0.11, 8.6 ± 0.6, 22.5 ± 0.5 & 52.1 ± 2.2) respectively for in-vehicle control, TMZ, PCI-24781 and TMZ + PCI-24781 treated U-118MG cells (Fig. 4C & D). To validate this apoptotic effect, we analyzed markers cleaved caspase 3 and cleaved poly ADP ribose polymerase (PARP), a DNA repair enzyme, in all aforementioned cell lines. We observed augmented cell death due to apoptosis in PCI-24781 treated cells and synergy with TMZ (Fig. 4C - E and Fig. S3A & S3B).

**Fig. 5:**
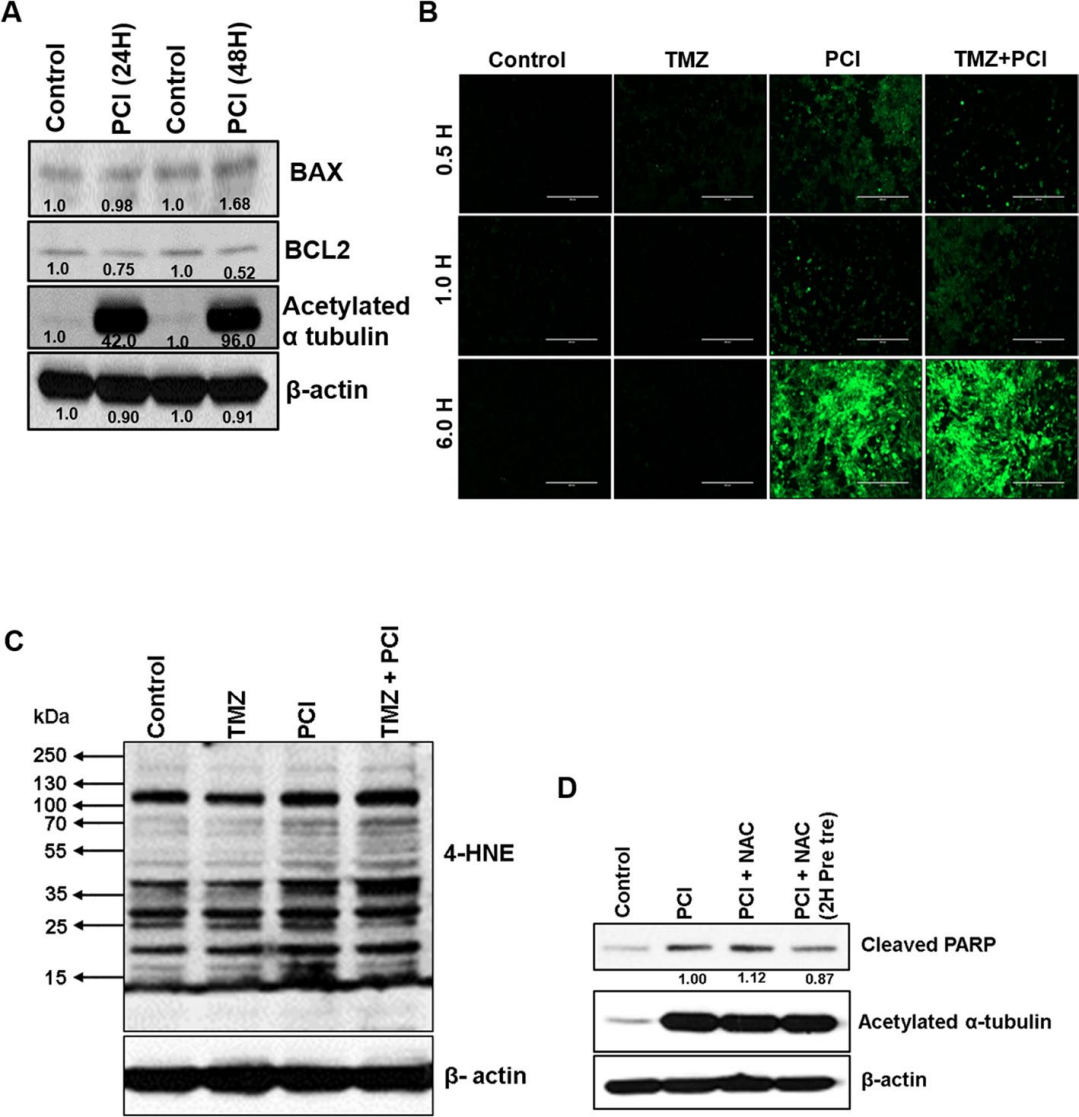
PCI-24781 displays a direct apoptotic effect in GBM cells. **A** U-118MG cells were treated with 1.25 μM PCI-24781 for 24 and 48 h, and cell lysates were analyzed for pro and anti-apoptotic markers by western blotting. **B-D** PCI-24781 induces ROS generation in GBM cells. **B** U-118MG cells were treated with vehicle control, TMZ, PCI-24781 or TMZ, and PCI-24781 combination for the indicated times. After incubation, media were removed, and cells rinsed with Krebs-Ringer buffer and stained with 10 μM DCF-DA at 37^•^C for 30 min in the dark. Finally, cells were washed in buffer, and fluorescent images were taken, and the representative images are shown. Scale bar 400 μm. **C** U-118MG cells were incubated with drugs for 6 h, and lysates were analyzed for lipid peroxidation marker 4-HNE by immunoblotting. **D** U-118MG cells were incubated with vehicle control, PCI-24781, PCI-24781 + NAC (15 mmol/L), or 2 h pre-treatment with NAC, followed by PCI-24781 for 48 h, and lysates were analyzed for apoptotic markers. Acetylation of α-tubulin serves as the marker for HDAC inhibitor PCI-24781 treatment. ROS- reactive oxygen species; NAC- N-acetylcysteine

HDAC inhibitors induce cell death in multiple ways, possibly by upregulating pro-apoptotic proteins, decreasing the anti-apoptotic proteins, and inducing reactive oxygen species (ROS) [33]. ROS production results in lipid peroxidation products like 4-hydroxynonenal (4-HNE), a biomarker of oxidative stress [34]. We checked pro-apoptotic protein BAX and anti-apoptotic protein BCL-2 expression in PCI-24781 treated U-118MG cells. PCI-24781 increased the expression of BAX while decreasing BCL2 expression at 48 h (Fig. 5A). Next, to test if PCI-24781 induced ROS production, followed by DNA damage resulting in cell death, we measured ROS production using 2′,7′-dichlorofluorescein diacetate (DCFDA) [35]. ROS induction was seen in PCI-24781-treated GBM cells within 30 min and peaked at 6 h (Fig. 5B). These results were further validated by immunoblotting with lipid peroxidation marker 4-HNE. Our immunoblotting results support ROS generation, and 4-HNE adduct formation in PCI-24781 treated GBM cells (Fig. 5C). To understand the consequence of ROS induction by PCI-24781 on cell death, U118-MG cells were treated with PCI-24781 in the presence and absence of ROS scavenger N-acetylcysteine (NAC) and the apoptotic marker cleaved PARP was analyzed. NAC (15 mmol/L) did not alter the extent of cell death, although two hours of NAC exposure before PCI-24781 was slightly protective (Fig. 5D). These results indicate that ROS production facilitates cell death but is not solely responsible for PCI-24781 mediated toxicity.

### Effect of PCI-24781 on DNA repair machinery

**Fig. 6:**
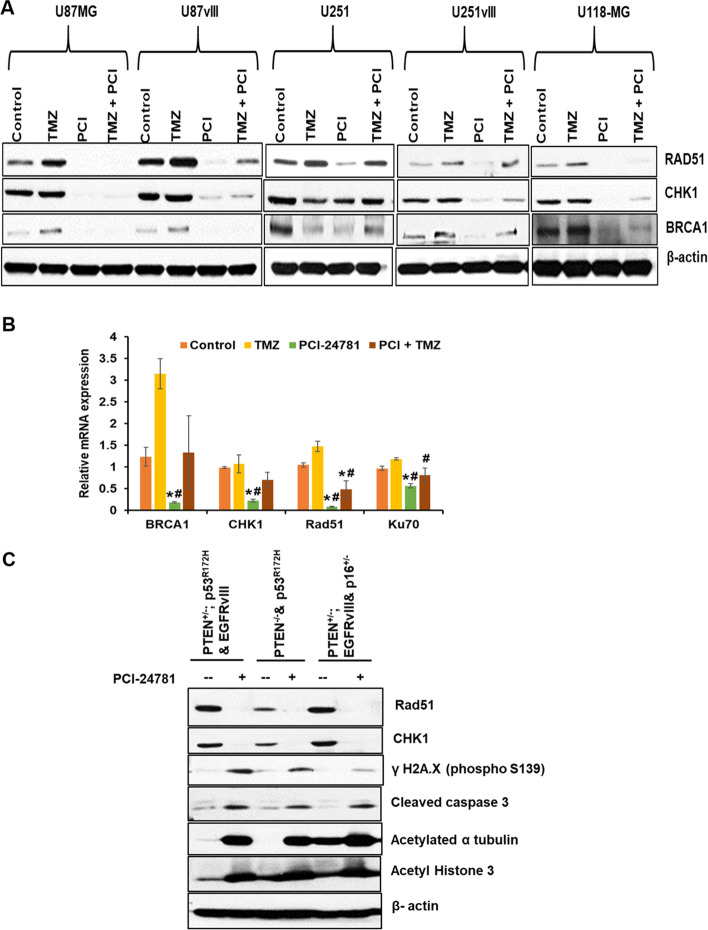
PCI-24781 decreases DNA repair machinery at the transcriptional level in GBM cells. **A** Cells were incubated with IC_25_ of TMZ, IC_25_ of PCI-24781 or combination for 48 h, and lysates were analyzed for RAD51, CHK1, and BRCA1 levels by immunoblotting. β-actin serves as a loading control. **B** Real-Time qRT-PCR for RNA expression of BRCA1, CHK1, RAD51, and Ku70 normalized to GAPDH in drug-treated (as given in (A)) U-118MG cells. The qPCR data is analyzed using the 2^-ΔΔCT^ method. ANOVA compared the ΔCt values between groups, and Tukey’s method was used to adjust for multiple comparisons. Results are given as fold change between groups with 95% confidence intervals. ‘*’ p < 0.01; ‘#’ *p* ≤ 0.01.”*” significantly different from vehicle control; “#” significantly different from TMZ. Data presented as mean +/− SD, experiments done in triplicate. **C** Mouse syngeneic cell lines were treated with 1.5 μM PCI-24781 for 48 h, and cell lysates were analyzed for DNA repair machinery and apoptotic marker by western blotting. ‘–‘ and ‘+’ signs indicate vehicle and PCI-24781 treatment, respectively

Pan-HDAC inhibitors panobinostat and vorinostat hyperacetylate nuclear HSP90, resulting in proteasomal degradation of DNA repair machinery proteins BRCA1, ATR, and CHK1 in breast cancer cells [9]. In contrast, Kachhap et al. demonstrated that downregulation of DNA repair genes BRCA1, CHK1, and RAD51 occurs at a transcriptional level upon HDAC inhibition [36]. Our western blot analysis showed that PCI-24781 potentially reduced the DNA repair machinery proteins RAD51, CHK1, and BRCA1 in various human GBM cells (Fig. 6A). To identify whether this degradation occurs at the transcriptional vs. the post-translational level, we performed Quantitative Real-Time-PCR on reverse-transcribed cDNA obtained from PCI-24781 treated U-118MG cells. We observed that PCI-24781 treatment significantly reduced the BRCA1, CHK1, RAD51, and Ku70 mRNA levels (*p* < 0.01) (Fig. 6B). We also validated the effect of PCI-24781 as a monotherapy to induce cell death and abrogate DNA repair protein in our in-house generated mouse syngeneic GBM cell lines. We observed that PCI-24781 reduced the expression of Rad51, CHK1 while activating γH2AX and caspase 3 (Fig. 6C). Our results suggest that PCI-24781 can induce cell death in syngeneic cell lines irrespective of the mutational status of p53, EGFRvIII, and p16 deletion.

### PCI-24781 induces DNA double-strand breaks in GBM cells

**Fig. 7:**
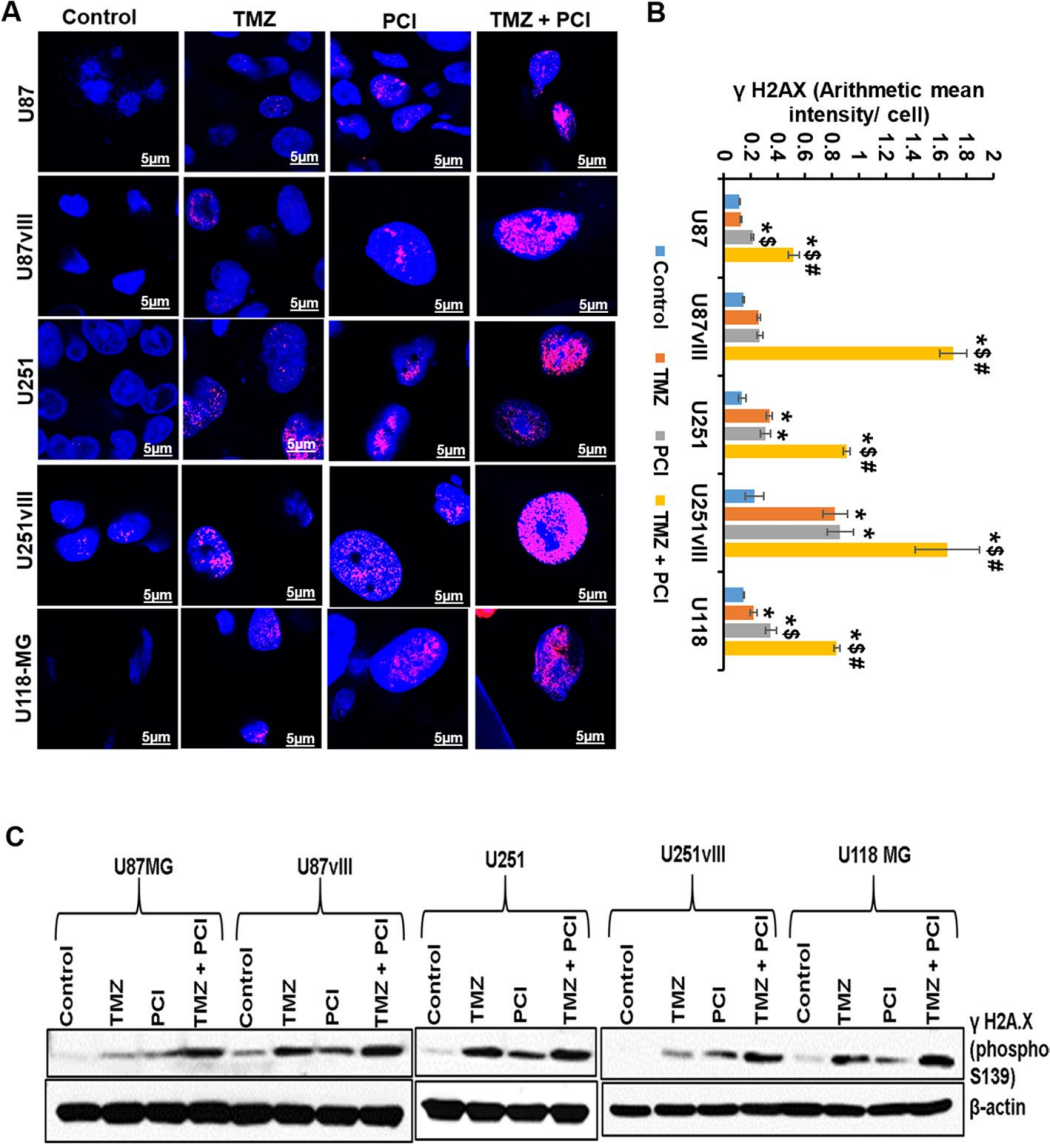
PCI-24781 induced DNA DSB (γH2AX foci) in GBM cells. **A** GBM cells were incubated with vehicle control, TMZ, PCI-24781, and combination for 48 h and probed with γH2AX (phospho S139) antibody, and nuclei counterstained with DAPI. Representative immunofluorescence images are shown. Scale Bar 5 μm. **B** Quantitative measurement of γH2AX staining represented as an arithmetic mean intensity per cell. Staining data were compared between control and treatment groups using one-way ANOVA. Pairwise comparisons between control and treatment groups were adjusted for multiple comparisons with Tukey’s method. “*” significantly different compared to vehicle control; ‘*’ *p* < 0.05; ‘$’ p < 0.001; ‘#’ p < 0.0001. “$” significantly different compared to TMZ; “#” significantly different compared to PCI-24781. **C** Immunoblot analysis of γH2AX (phospho S139) levels in vehicle or drug-treated GBM cells. DNA DSB- DNA double-strand break

H2AX is the key enzyme involved in the DNA damage repair process and is phosphorylated at the serine-139 during DNA damage [37]. We analyzed the phospho-histone H2AX (Ser139) staining to indicate DNA double-strand breaks (DSBs) in TMZ and PCI-24781 treated GBM cells. We used confocal microscopy to identify the γ-H2AX foci formation upon drug treatment. As expected, the alkylating agent TMZ induced DNA damage in GBM cells (Fig. 7A). Interestingly, PCI-24781 alone also induced DNA damage, as evidenced by cells positive for γ-H2AX and enlarged nuclei (Fig. 7A and Fig. S4). Our quantitative analysis revealed that PCI-24781 treatment resulted in 83, 86, 130, 283, and 136% γ-H2AX arithmetic mean intensity increases in U87, U87EGFRvIII, U251, U251EGFRvIII, and U-118MG cells respectively when compared to untreated control (Fig. 7B). This effect was significantly augmented by the addition of TMZ (*p* < 0.001) (Fig. 7B). These results were further confirmed by γ-H2AX immunoblotting (Fig. 7C).

### PCI-24781 is effective in MGMT expressing GBM orthografts and a genetically engineered mouse model for GBM

**Fig. 8:**
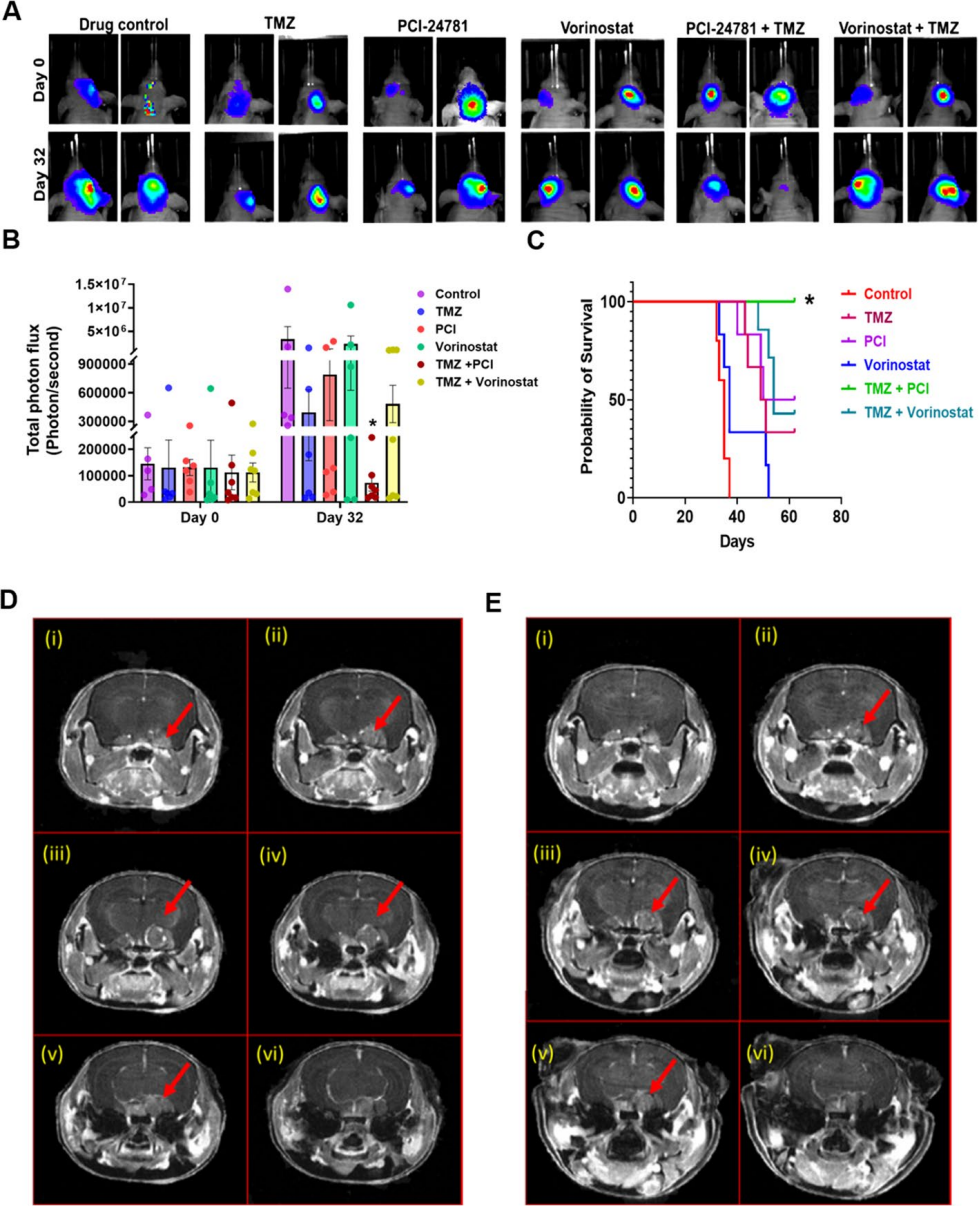
PCI-24781 and TMZ in combination decrease tumor burden in vivo. **A** Animals treated with both drugs had more significant decreases in tumor burden. U-118MG luciferase transfected cells (1X10^5 cells in 3 μl of PBS) were intracranially injected into 4 to 6 week-old mice. After 10 days, BLI measured tumor growth, and animals were randomized into 6 groups and treated with vehicle control, TMZ (25 mg/kg BW), PCI-24781 (12.5 mg/kg BW, BID), vorinostat (100 mg/kg/BW), PCI-24781+ TMZ or vorinostat + TMZ for 5 days a week. Qualitative BLI imaging is shown in (**A**), and quantitative measurements (total photon flux, photons/second) of the BLI from the tumor is shown in (**B**). ‘*‘*p* < 0.05 .“*” significantly different compared to vehicle control. BLI- bioluminescence imaging. Data presented as mean +/− SEM and analyzed using the natural log of photon levels on Day 32 compared to control and treatment groups using one-way ANOVA. Pairwise comparisons were adjusted for multiple comparisons with Dunnett’s method. **C** PCI-24781 improved survival over vorinostat, and PCI + TMZ significantly increased overall survival. Overall survival was estimated using the Kaplan-Meier method, and groups were compared using the log-rank test. Mice alive at the end of the study were treated as censored. ‘*‘*p* < 0.0001. “*” significantly different compared to vehicle control. **D** & **E** PCI-24781 decreases tumor burden in the GBM mouse model. MRI was done on a 7 Tesla scanner. (Bruker PharmaScan, Billerica, MA) operated by ParaVision 7 with a quadrature RF coil for signal transmission and reception. MRI on GBM mouse (*PEPG* - PTEN^flox/+^; EGFRvIII+; p16^Flox/−^ & GFAP Cre +). **D**; (i)-(vi) shows the pre-treatment axial T1post images of a mouse brain in rostral-caudal direction. **E** (i)-(vi) Axial T1post images acquired after 4 weeks of 5-days weekly PCI-24781 (12.5 mg/kg BW, BID, PO)

Although PCI-24781 decreases the viability of human GBM cell lines in vitro, the in vivo system presents a restrictive drug accessibility barrier via the BBB for brain tumors. To study the efficacy of PCI-24781 in vivo, we used U-118MG orthografts. We intracranially injected U-118MG cells transfected with GFP-luciferase into athymic nude mice. After a week, tumor growth was measured by substrate CycLuc1 i.p. injection, followed by Bioluminescence imaging (BLI). Based on tumor size, animals were randomized to treat i) vehicle control, ii) TMZ, iii) PCI-24781, iv) vorinostat, or v) combination TMZ + PCI or  vi) TMZ + vorinostat. IVIS imaging showed the tumor growth decreased by 86, 72, 17, 97 and 80% in TMZ, PCI-24781, vorinostat, TMZ + PCI and TMZ + vorinostat groups, respectively compared to control (Fig. 8A & B). TMZ + PCI significantly decreased the tumor burden compared to control (*p* < 0.05) (Fig. 8A & B) and significantly increased the OS (*p* < 0.0001) (Fig. 8C), supporting BBB permeability of PCI-24781 and synergy with TMZ. TMZ + PCI-24781 treated mice were sacrificed after 92 days, and vehicle-treated, and other drug-treated group mice were sacrificed when they were weak. Figure 8D (Fig. S5) shows the pre-treatment T1 post-contrast images of a *PEPG* (PTEN^flox/+^; EGFRvIII+; p16^Flox/−^ & GFAP Cre +) 4.5 month old mouse. Tumor is close to the hypothalamus and thalamus, denoted by arrows (Fig. 8D). The T1 post-contrast images after 4 weeks of PCI-24781 are shown in Fig. 8E. Tumor volume decreased from 7.1 mm^3^ to 5.2 mm^3^ on ROI analysis, a 26.4% decrease.

## Discussion

GBM has a dismal 5-year survival of 10% [38]. Treatment includes surgery, RT, and TMZ [38]. Response to TMZ is curtailed by DNA repair mechanisms rendering alkylators less effective [39]. Unattended DNA DSBs can lead to genomic instability and cell death. There are two major pathways by which DNA DSB repair occurs: Homology–directed repair (HDR/HR) and non-homologous end-joining (NHEJ). TMZ is an alkylator that induces three common DNA lesions N^3^-methyladenine (N^3^-meA), N^7^-methylguanine (N^7^-meG), and O^6^-methylguanine (O^6^-meG) [40]. N^3^-meA and N^7^-meG are primarily repaired by the BER pathway, while O^6^-meG is repaired by MGMT [40]. Though much clinical focus is on MGMT methylation, TMZ-induced O^6^-meG DNA damage is minimal (~ 5 to 10%), and the remaining ~ 90% are at N^3^-meA and N^7^-meG [41]. TMZ resistance is also caused by alkylpurine-DNA-N-glycosylase (APNG), a BER enzyme, in T98G GBM cells [42]. Silencing of BER enzyme apyrimidinic/apurinic endonuclease/redox factor-1 sensitized T98G cells to TMZ [43]. Adimoolam et al. provided direct evidence for PCI-24781, which decreased HR activity in Chinese hamster ovary cells, possibly by downregulating RAD51 [44]. Additionally, HDAC inhibition increased the synthetic lethality of the PARP inhibitor veliparib/ABT-888 in prostate cancer cells by inhibiting HR DNA repair signaling [45].

This study used an agnostic approach to drug identification by starting with an in silico method applying the tool CMap, to identify drugs for repurposing based on their genetic profiles for GBM. Utilizing the GEPIA database and further scrutinizing inhibitor selectivity, we selected PCI-24781/abexinostat, which effectively targets GBM cells. Our GEPIA database analysis revealed that HDAC11 is significantly downregulated in GBM. Tumor growth rates of murine lymphoma (EL4) and pancreatic adenocarcinoma cell lines (Panco) were significantly higher in HDAC11 knock out C57BL/6 mice [46], and myeloid-derived suppressor cells (MDSCs) isolated from HDAC11 knock out mice seem more suppressive than the WT MDSCs. Moreover, loss of HDAC11 function induces the immunosuppressive interleukin-10 [46]. PCI-24781 does not inhibit HDAC11, as do belinostat and trichostatin A. Certainly, PCI-24781 displayed strong cytotoxic effects on neuroblastoma cell lines with an IC_50_ of less than 200 nanomolar while other pan HDAC inhibitors (sodium butyrate, SAHA and valproic acid) had IC_50_ values in the ranges of micromolar to millimolar [47].

We evaluated the anticancer activities of PCI-24781 alone and with TMZ. PCI-24781 significantly inhibited proliferation and tumorigenicity while augmenting apoptosis via downregulation of DNA repair machinery proteins (BRCA1, RAD51, CHK1) in GBM cells. In vivo, PCI-24781 effectively crossed the BBB and efficiently decreased tumor burden in orthotopic GBM xenografts alone and with TMZ.

In prior studies, vorinostat displayed anti-proliferative effects in glioma cells in a p53 independent manner [48]. Our MTT assays also support PCI-24781 GBM cell growth inhibition irrespective of p53 status. Furthermore, its anti-tumorigenic potential was unaffected by MGMT and EGFRvIII over-expression.

Vorinostat induces ROS production in a concentration-dependent manner, and [49] vorinostat-mediated cell death was inhibited by 50% with antioxidant NAC treatment [49]. Our studies showed PCI-24781 induced ROS production in GBM cells, but NAC treatment did not attenuate GBM cell death, suggesting ROS generation is not entirely responsible for PCI-24781 induced cytotoxicity as it may be with vorinostat.

Hyperacetylation of HSP90 changes its chaperone function and facilitates polyubiquitylation and proteasomal degradation of HSP90 target proteins [8]. Pan-HDAC inhibitors panobinostat and vorinostat hyperacetylate nuclear HSP90, which degrades DNA repair machinery proteins BRCA1, ATR, and CHK1 in breast cancer cells [9]. However, downregulation of DNA repair genes BRCA1, CHK1, and RAD51 occurs at the transcriptional level through a decrease in the recruitment of transcription factor E2F1 upon HDAC inhibition [36]. GEPIA database analysis revealed RAD51, CHK1, BRCA1, and Ku70 are upregulated in GBM and that higher expression of RAD51 [50] and BRCA1 [51] were associated with shorter survival. Our western blot analysis on PCI-24781 treated GBM cell lysates showed decreased expression of BRCA1, CHK1, and RAD51. Further, quantitative real-time-PCR analysis on BRCA1, CHK1, and RAD51 in PCI-24781 treated GBM cells revealed that silencing occurs at the transcriptional level.

DNA DSBs in chromatin induce phosphorylation at serine 139 of the histone H2A variant, H2AX, producing γ-H2AX [52]. γ-H2AX generation is essential to recruit several DNA repair proteins, which inhibit cell cycle progression by regulating cell cycle checkpoints [53]. HDAC inhibitors added to radiation increase the duration of γ-H2AX and radiosensitivity of prostate and melanoma cells [53]. Of note, BRCA1 and BRCA2 proteins colocalize with RAD51, essential for HR repair of DNA DSBs [54]. PCI-24781 treatment decreased RAD51 levels and the HR activity in Chinese hamster ovary cells [44]. Further, HDAC inhibition increased chemosensitivity of prostate cancer to the PARP inhibitor veliparib/ABT-888 by inhibiting HR DNA repair signaling [45]. A recent study shows that increased DNA damage is associated with enlarged nuclei [55]. Our studies showed that PCI-24781 decreases the RAD51 and BRCA1 levels and enhances γ-H2AX formation, nuclei enlargement, and apoptotic effects of TMZ.

Deutsch et al. demonstrated that (^14^C)- PCI-24781 was found in the cerebrum, cerebellum, plasma, and all tissues after IV administration; evidence for BBB penetration [56], further supported by our BLI data. Orally administered PCI-24781 plus TMZ treatment potentially inhibited the growth of intracranial U-118MG GBM xenografts compared to vorinostat plus TMZ. Further, our MRI of a PCI-24781 treated GBM mouse model also supports its anti-tumorigenic potential. Intraperitoneal vorinostat decreased tumor burden in a murine glioma model but only increased median survival by 8 days [48]. Two other preclinical studies with vorinostat displayed better efficacy; however, they injected vorinostat intracranially [57, 58], another route that does not mimic normal human drug administration. Thus, ineffective drug distribution may be one reason behind the limited clinical efficacy of vorinostat in GBM patients. However, in our study, lower concentrations of PCI-24781 (12.5 mg/kg BW, two times per day, 5 days/week, given via oral gavage), provided greater efficacy with less toxicity. While many HDAC inhibitors are cardiotoxic, leading to ventricular arrhythmias and QT/QTc prolongation [59], PCI-24781 was well tolerated in clinical study, with only one patient experiencing grade 3 QTc prolongation phase II PCI-24781 [59].

## Conclusion

Utilizing in silico analysis to efficiently decrease drug screening time, we identified PCI-24781 based on its unique inhibitor selectivity and apoptotic potential. PCI-24781 displayed strong anti-proliferative effects on GBM cells in vitro, irrespective of the mutational profile. Additionally, it enhanced TMZ-induced apoptosis, possibly by increasing γ-H2AX formation through the downregulation of DNA DSB repair proteins. In vivo, PCI-24781 decreased the tumor burden of GBM orthografts and in a GBM mouse model. These exciting results provide the rationale for a clinical trial combining PCI-24781 with TMZ to improve survival in all GBM patients, agnostic of mutational profile.

## Supplementary Information

**Additional file 1: Supplementary methods**. **Table S1** Drugs common across the four datasets. **Table S2** Inhibitor selectivity. **Table S3** PCI-24781 inhibits GBM cell viability. **Table S4** List of antibodies used in this study. **Figure S1**: PCI-24781 decreases the viability of MGMT expressing human U-118MG and EGFRvIII, expressing mouse syngeneic GBM cells. **Figure S2**: PCI-24781 + TMZ combination significantly decreases the tumorigenicity of EGFRvIII+, p16^Flox/Flox^, GFAP Cre + mouse syngeneic GBM cells. **Figure S3**: PCI-24781 shows strong synergistic effects with TMZ in GBM cells. **Figure S4**: PCI-24781 induces nuclear enlargement in U-118MG cells. **Figure S5**: Genotyping of GEM GBM model.

## Data Availability

The datasets used and/or analyzed during this study are freely available.

## Abbreviations

BBB: Blood-brain barrier
BER: Base Excision Repair
BLI: Bioluminescence imaging
CMap: Connectivity Map
GBM: Glioblastoma
GEO: Gene Expression Omnibus
HAT: Histone acetyltransferase
HDAC: Histone deacetylase
HDR/HR: Homology–directed repair
4-HNE: 4-hydroxynonenal
HSP90: Heat shock protein 90
MGMT: O^6^-methylguanine-DNA- methyltransferase
MMR: Mismatch repair
MRI: Magnetic resonance imaging
NAC: N-acetylcysteine
N^7^-meG: N^7^-methylguanine
N^3^-meA: N^3^-methyladenine
NHEJ: Non-homologous end-joining
O^6^-meG: O^6^-methylguanine
OS: Overall survival
RT: Radiation therapy
TMZ: Temozolomide

## References

[1] Ostrom QT, Gittleman H, Fulop J, Liu M, Blanda R, Kromer C (2015). CBTRUS statistical report: primary brain and central nervous system tumors diagnosed in the United States in 2008–2012. Neuro-Oncology.

[2] Stupp R, Mason WP, van den Bent MJ, Weller M, Fisher B, Taphoorn MJ (2005). Radiotherapy plus concomitant and adjuvant temozolomide for glioblastoma. N Engl J Med.

[3] Hegi ME, Diserens AC, Gorlia T, Hamou MF, de Tribolet N, Weller M (2005). MGMT gene silencing and benefit from temozolomide in glioblastoma. N Engl J Med.

[4] Erasimus H, Gobin M, Niclou S, Van Dyck E (2016). DNA repair mechanisms and their clinical impact in glioblastoma. Mutat Res Rev Mutat Res.

[5] Annovazzi L, Mellai M, Schiffer D. Chemotherapeutic drugs: DNA damage and repair in Glioblastoma. Cancers (Basel). 2017;9(6). 10.3390/cancers9060057. 10.3390/cancers9060057 PMC5483876 28587121

[6] Jenuwein T, Allis CD (2001). Translating the histone code. Science.

[7] Lindemann RK, Newbold A, Whitecross KF, Cluse LA, Frew AJ, Ellis L (2007). Analysis of the apoptotic and therapeutic activities of histone deacetylase inhibitors by using a mouse model of B cell lymphoma. Proc Natl Acad Sci U S A.

[8] Bali P, Pranpat M, Bradner J, Balasis M, Fiskus W, Guo F (2005). Inhibition of histone deacetylase 6 acetylates and disrupts the chaperone function of heat shock protein 90: a novel basis for antileukemia activity of histone deacetylase inhibitors. J Biol Chem.

[9] Ha K, Fiskus W, Choi DS, Bhaskara S, Cerchietti L, Devaraj SG (2014). Histone deacetylase inhibitor treatment induces 'BRCAness' and synergistic lethality with PARP inhibitor and cisplatin against human triple negative breast cancer cells. Oncotarget.

[10] de Andrade PV, Andrade AF, de Paula Queiroz RG, Scrideli CA, Tone LG, Valera ET (2016). The histone deacetylase inhibitor PCI-24781 as a putative radiosensitizer in pediatric glioblastoma cell lines. Cancer Cell Int.

[11] Bangert A, Hacker S, Cristofanon S, Debatin KM, Fulda S (2011). Chemosensitization of glioblastoma cells by the histone deacetylase inhibitor MS275. Anti-Cancer Drugs.

[12] Staberg M, Michaelsen SR, Rasmussen RD, Villingshoj M, Poulsen HS, Hamerlik P (2017). Inhibition of histone deacetylases sensitizes glioblastoma cells to lomustine. Cell Oncol (Dordr).

[13] Li ZY, Li QZ, Chen L, Chen BD, Wang B, Zhang XJ (2016). Histone Deacetylase inhibitor RGFP109 overcomes Temozolomide resistance by blocking NF-kappaB-dependent transcription in Glioblastoma cell lines. Neurochem Res.

[14] Lee DH, Ryu HW, Won HR, Kwon SH (2017). Advances in epigenetic glioblastoma therapy. Oncotarget.

[15] Wu Y, Dong L, Bao S, Wang M, Yun Y, Zhu R (2016). FK228 augmented temozolomide sensitivity in human glioma cells by blocking PI3K/AKT/mTOR signal pathways. Biomed Pharmacother.

[16] Ghiaseddin A, Reardon D, Massey W, Mannerino A, Lipp ES, Herndon JE (2018). Phase II study of Bevacizumab and Vorinostat for patients with recurrent World Health Organization grade 4 malignant Glioma. Oncologist.

[17] Friday BB, Anderson SK, Buckner J, Yu C, Giannini C, Geoffroy F (2012). Phase II trial of vorinostat in combination with bortezomib in recurrent glioblastoma: a north central cancer treatment group study. Neuro-Oncology.

[18] Lee P, Murphy B, Miller R, Menon V, Banik NL, Giglio P (2015). Mechanisms and clinical significance of histone deacetylase inhibitors: epigenetic glioblastoma therapy. Anticancer Res.

[19] Iwamoto FM, Lamborn KR, Kuhn JG, Wen PY, Yung WK, Gilbert MR (2011). A phase I/II trial of the histone deacetylase inhibitor romidepsin for adults with recurrent malignant glioma: north American brain tumor consortium study 03-03. Neuro-Oncology.

[20] Lamb J, Crawford ED, Peck D, Modell JW, Blat IC, Wrobel MJ (2006). The connectivity map: using gene-expression signatures to connect small molecules, genes, and disease. Science.

[21] Joy A, Ramesh A, Smirnov I, Reiser M, Misra A, Shapiro WR (2014). AKT pathway genes define 5 prognostic subgroups in glioblastoma. PLoS One.

[22] Birks DK, Donson AM, Patel PR, Sufit A, Algar EM, Dunham C (2013). Pediatric rhabdoid tumors of kidney and brain show many differences in gene expression but share dysregulation of cell cycle and epigenetic effector genes. Pediatr Blood Cancer.

[23] Griesinger AM, Birks DK, Donson AM, Amani V, Hoffman LM, Waziri A (2013). Characterization of distinct immunophenotypes across pediatric brain tumor types. J Immunol.

[24] Mangiola A, Saulnier N, De Bonis P, Orteschi D, Sica G, Lama G (2013). Gene expression profile of glioblastoma peritumoral tissue: an ex vivo study. PLoS One.

[25] Ritchie ME, Phipson B, Wu D, Hu Y, Law CW, Shi W (2015). Smyth GK: limma powers differential expression analyses for RNA-sequencing and microarray studies. Nucleic Acids Res.

[26] Pilarczyk M, Najafabadi MF, Kouril M, Vasiliauskas J, Niu W, Shamsaei B, et al. Connecting omics signatures of diseases, drugs, and mechanisms of actions with iLINCS. bioRxiv. 2019:826271. 10.1101/826271.

[27] Kuhn M, von Mering C, Campillos M, Jensen LJ, Bork P (2008). STITCH: interaction networks of chemicals and proteins. Nucleic Acids Res.

[28] TC Chou NM (2005). CompuSyn for drug combinations: PC software and User’s guide: a computer program for quantitation of synergism and antagonism in drug combinations, and the determination of IC50 and ED50 and LD50 values.

[29] Vengoji R, Macha MA, Nimmakayala RK, Rachagani S, Siddiqui JA, Mallya K (2019). Afatinib and Temozolomide combination inhibits tumorigenesis by targeting EGFRvIII-cMet signaling in glioblastoma cells. J Exp Clin Cancer Res.

[30] Torres MP, Rachagani S, Souchek JJ, Mallya K, Johansson SL, Batra SK (2013). Novel pancreatic cancer cell lines derived from genetically engineered mouse models of spontaneous pancreatic adenocarcinoma: applications in diagnosis and therapy. PLoS One.

[31] Tang Z, Li C, Kang B, Gao G, Li C, Zhang Z (2017). GEPIA: a web server for cancer and normal gene expression profiling and interactive analyses. Nucleic Acids Res.

[32] Nagane M, Narita Y, Mishima K, Levitzki A, Burgess AW, Cavenee WK (2001). Human glioblastoma xenografts overexpressing a tumor-specific mutant epidermal growth factor receptor sensitized to cisplatin by the AG1478 tyrosine kinase inhibitor. J Neurosurg.

[33] Schrump DS (2009). Cytotoxicity mediated by histone deacetylase inhibitors in cancer cells: mechanisms and potential clinical implications. Clin Cancer Res.

[34] Liu W, Porter NA, Schneider C, Brash AR, Yin H (2011). Formation of 4-hydroxynonenal from cardiolipin oxidation: Intramolecular peroxyl radical addition and decomposition. Free Radic Biol Med.

[35] Muniyan S, Chou YW, Tsai TJ, Thomes P, Veeramani S, Benigno BB (2015). p66Shc longevity protein regulates the proliferation of human ovarian cancer cells. Mol Carcinog.

[36] Kachhap SK, Rosmus N, Collis SJ, Kortenhorst MS, Wissing MD, Hedayati M (2010). Downregulation of homologous recombination DNA repair genes by HDAC inhibition in prostate cancer is mediated through the E2F1 transcription factor. PLoS One.

[37] Kuo LJ, Yang LX (2008). Gamma-H2AX - a novel biomarker for DNA double-strand breaks. In Vivo.

[38] Lieberman F (2017). Glioblastoma update: molecular biology, diagnosis, treatment, response assessment, and translational clinical trials. F1000Res.

[39] Chen X, Zhang M, Gan H, Wang H, Lee JH, Fang D (2018). A novel enhancer regulates MGMT expression and promotes temozolomide resistance in glioblastoma. Nat Commun.

[40] Fu D, Calvo JA, Samson LD (2012). Balancing repair and tolerance of DNA damage caused by alkylating agents. Nat Rev Cancer.

[41] Lee SY (2016). Temozolomide resistance in glioblastoma multiforme. Genes Dis.

[42] Agnihotri S, Gajadhar AS, Ternamian C, Gorlia T, Diefes KL, Mischel PS (2012). Alkylpurine-DNA-N-glycosylase confers resistance to temozolomide in xenograft models of glioblastoma multiforme and is associated with poor survival in patients. J Clin Invest.

[43] Montaldi AP, Godoy PR, Sakamoto-Hojo ET (2015). APE1/REF-1 down-regulation enhances the cytotoxic effects of temozolomide in a resistant glioblastoma cell line. Mutat Res Genet Toxicol Environ Mutagen.

[44] Adimoolam S, Sirisawad M, Chen J, Thiemann P, Ford JM, Buggy JJ (2007). HDAC inhibitor PCI-24781 decreases RAD51 expression and inhibits homologous recombination. Proc Natl Acad Sci U S A.

[45] Yin L, Liu Y, Peng Y, Peng Y, Yu X, Gao Y (2018). PARP inhibitor veliparib and HDAC inhibitor SAHA synergistically co-target the UHRF1/BRCA1 DNA damage repair complex in prostate cancer cells. J Exp Clin Cancer Res.

[46] Sahakian E, Powers JJ, Chen J, Deng SL, Cheng F, Distler A (2015). Histone deacetylase 11: a novel epigenetic regulator of myeloid derived suppressor cell expansion and function. Mol Immunol.

[47] Sholler GS, Currier EA, Dutta A, Slavik MA, Illenye SA, Mendonca MC (2013). PCI-24781 (abexinostat), a novel histone deacetylase inhibitor, induces reactive oxygen species-dependent apoptosis and is synergistic with bortezomib in neuroblastoma. J Cancer Ther Res.

[48] Yin D, Ong JM, Hu J, Desmond JC, Kawamata N, Konda BM (2007). Suberoylanilide hydroxamic acid, a histone deacetylase inhibitor: effects on gene expression and growth of glioma cells in vitro and in vivo. Clin Cancer Res.

[49] Ruefli AA, Ausserlechner MJ, Bernhard D, Sutton VR, Tainton KM, Kofler R (2001). The histone deacetylase inhibitor and chemotherapeutic agent suberoylanilide hydroxamic acid (SAHA) induces a cell-death pathway characterized by cleavage of bid and production of reactive oxygen species. Proc Natl Acad Sci U S A.

[50] Balbous A, Cortes U, Guilloteau K, Rivet P, Pinel B, Duchesne M (2016). A radiosensitizing effect of RAD51 inhibition in glioblastoma stem-like cells. BMC Cancer.

[51] Rasmussen RD, Gajjar MK, Tuckova L, Jensen KE, Maya-Mendoza A, Holst CB (2016). BRCA1-regulated RRM2 expression protects glioblastoma cells from endogenous replication stress and promotes tumorigenicity. Nat Commun.

[52] Kinner A, Wu W, Staudt C, Iliakis G (2008). Gamma-H2AX in recognition and signaling of DNA double-strand breaks in the context of chromatin. Nucleic Acids Res.

[53] Geng L, Cuneo KC, Fu A, Tu T, Atadja PW, Hallahan DE (2006). Histone deacetylase (HDAC) inhibitor LBH589 increases duration of gamma-H2AX foci and confines HDAC4 to the cytoplasm in irradiated non-small cell lung cancer. Cancer Res.

[54] Zhang H, Tombline G, Weber BL (1998). BRCA1, BRCA2, and DNA damage response: collision or collusion?. Cell.

[55] Raghavan S, Baskin DS, Sharpe MA. A "clickable" probe for active MGMT in Glioblastoma demonstrates two discrete populations of MGMT. Cancers (Basel). 2020;12(2). 10.3390/cancers12020453. 10.3390/cancers12020453 PMC7072665 32075134

[56] Deutsch E, Moyal EC, Gregorc V, Zucali PA, Menard J, Soria JC, Kloos I, Hsu J, Luan Y, Liu E, Vezan R, Graef T, Rivera S (2017). A phase 1 dose-escalation study of the oral histone deacetylase inhibitor abexinostat in combination with standard hypofractionated radiotherapy in advanced solid tumors. Oncotarget.

[57] Eyupoglu IY, Hahnen E, Buslei R, Siebzehnrubl FA, Savaskan NE, Luders M (2005). Suberoylanilide hydroxamic acid (SAHA) has potent anti-glioma properties in vitro, ex vivo and in vivo. J Neurochem.

[58] Ugur HC, Ramakrishna N, Bello L, Menon LG, Kim SK, Black PM (2007). Continuous intracranial administration of suberoylanilide hydroxamic acid (SAHA) inhibits tumor growth in an orthotopic glioma model. J Neuro-Oncol.

[59] Evens AM, Balasubramanian S, Vose JM, Harb W, Gordon LI, Langdon R (2016). A phase I/II multicenter, open-label study of the Oral histone Deacetylase inhibitor Abexinostat in relapsed/refractory lymphoma. Clin Cancer Res.

