# Supplementary material for: Differential gene expression-based connectivity mapping identified novel drug candidate and improved Temozolomide efficacy for Glioblastoma
Source: J Exp Clin Cancer Res. 2021 Oct 25;40:335. doi: 10.1186/s13046-021-02135-x (PMC8543939; doi:10.1186/s13046-021-02135-x)
Supplement: Supplementary file 1 — Additional file 1: Supplementary methods. Table S1 Drugs common across the four datasets. Table S2 Inhibitor selectivity. Table S3 PCI-24781 inhibits GBM cell viability. Table S4 List of antibodies used in this study. Figure S1: PCI-24781 decreases the viability of MGMT expressing human U-118MG and EGFRvIII, expressing mouse syngeneic GBM cells. Figure S2: PCI-24781 + TMZ combination significantly decreases the tumorigenicity of EGFRvIII+, p16Flox/Flox, GFAP Cre + mouse syngeneic GBM cells. Figure S3: PCI-24781 shows strong synergistic effects with TMZ in GBM cells. Figure S4: PCI-24781 induces nuclear enlargement in U-118MG cells. Figure S5: Genotyping of GEM GBM model. [file 13046_2021_2135_MOESM1_ESM.zip › Supplementary methods. clean R1.docx]

**Supplementary methods**

**GBM cell lines and cell cultures.** Human GBM U87 and U87 cell lines transfected with EGFRvIII were a kind gift from Dr. Webster K. Cavenee (University of California San Diego, USA) and U251 and U251 cells transfected with EGFRvIII were a generous gift from Dr. Amyn A. Habib (University of Texas Southwestern Medical Center, Dallas, TX USA). U-118MG cells were purchased from ATCC® cell lines. All the human and mouse syngeneic cell lines were cultured in Dulbecco’s modified Eagle medium (DMEM) supplemented with 10% fetal bovine serum (FBS) and penicillin (100 units/ml) and streptomycin (0.25 µg/ml). Tetracycline (Tet) - inducible promoter drives the EGFRvIII expression in U251 EGFRvIII cells [1]. Cell line validation was done at the University of Arizona genetics core, Tucson, AZ. USA, by PCR-based short-tandem repeat (STR) analysis.

***In vitro* tumorigenic assay.** In vitro tumorigenic/clonogenic or colony formation assay was done as described earlier with slight modifications [1, 2]. Briefly, 2 x 10^^3^ cells were seeded in a six-well plate containing complete DMEM. Following overnight incubation, cells were treated with either vehicle control (0.02% DMSO), PCI-24781, TMZ, or TMZ + PCI-24781. After 48 hours, cells were washed once with PBS and allowed to grow in a complete medium for two weeks, and colonies were stained with crystal violet. Colonies were dissolved in 10% acetic acid, and percentage colony formation was calculated from the absorbance measured at 595 nm using a plate reader [2].

**Apoptosis assay.** Apoptosis was measured as described earlier with slight modifications[3]**.** Briefly, 0.8 x 10^^6^ U-118MG cells were seeded in a 60mm dish. After overnight incubation at 37^•^ C in a CO_2_ incubator, cells were treated with vehicle control (0.02% DMSO) or drugs for 48 hours in 10% DMEM. The percentage of early and late apoptotic cells were measured by staining the cells with Cy™5 Annexin V (BD Biosciences, USA) and propidium iodide (Sigma-Aldrich, USA) solution, followed by the analysis of the cells using Flow cytometry.

**Quantitative Real-Time-PCR.** Total RNA was isolated using RNeasy kit (QIAGEN, USA), and complementary DNA (cDNA) was synthesized as described by the manufacturer’s instructions (iScript Reverse Transcription Supermix for RT-qPCR, BIO-RAD, USA). Real-Time-PCR was done on BIO-RAD CFX connect ^TM^ Real-Time system using the following primer sequences; RAD51 forward, 5′- TCTCTGGCAGTGATGTCCTGGA-3′; RAD51 reverse, 5′- TAAAGGGCGGTGGCACTGTCTA-3′ [4]; BRCA1 forward, 5′-GCGTCCCCTCACAAATAAAT-3′; BRCA1 reverse, 5′-CTTGACCATTCTGCTCCGTT-3’ [5]; GAPDH forward, 5′-TCAAGAAGGTGGTGAAGCAG-3′; GAPDH reverse, 5′-AAAGGTGGAGGAGTGGGTGT-3′ [5]; CHK1 forward, 5′- GGTGCCTATGGAGAAGTTCAA-3′; CHK1 reverse, 5′- TCTACGGCACGCTTCATATC-3′ [6]; Ku70 forward, 5′- CCAAGACCCGGACCTTTAATAC -3′; Ku70 reverse, 5′- AGTATAATCTGACGACTCCCATAGA -3 [7]′. Each sample and control was run in triplicate for each assay under similar conditions and normalized with GAPDH. Data were calculated using the 2^-ΔΔCT^ method [8].

**Fluorescence immunostaining.** Cells were grown on glass coverslips (50,000 cells/ 12 well plate), and drug treatment applied for 48 hours. Following, cells were fixed in ice-cold methanol, and blocking was done with 10% normal goat serum (NGS) for 1 hour at room temperature. Primary antibody (Rabbit phosphor-histone H2A.X (Ser139) antibody (Cell Signaling Technology, USA; Cat # 2577)) was used at 1:400 dilution overnight. After three washes with PBS (5 minutes each), we analyzed the γ-H2AX foci with goat anti-rabbit Alexa Fluor 568 (Invitrogen) and incubated them at 37^•^ C for 1 hour. Next, the slides were rewashed with PBS three times, then allowed to dry in the dark for half an hour and mounted with DAPI Fluor mount-G ® (Southern Biotech, Birmingham, AL, USA). Fluorescent images were captured using a Carl Zeiss microscope, and γ-H2AX arithmetic mean index per cell was quantified using ZEN 2.3 lite (blue edition) software, Carl Zeiss Microscopy GmbH, 2011 (Carl-Zeiss-Promenade 10 Jena, Germany).

**Western blotting.** Cell lysate preparation and protein estimation were done as described earlier [9]. Proteins (30 or 40 µg/ well) were resolved on 10% - 12% sodium dodecyl sulfate (SDS) - polyacrylamide gel electrophoresis (PAGE). They were then transferred onto polyvinylidene difluoride (PVDF) membranes by electroblotting. Membranes were washed with PBS containing 0.1% Tween 20 (PBST) and blocked with PBST containing 5% nonfat dry milk for 1 hour. After blocking, membranes were incubated with primary antibodies (1: 1000 dilutions in PBS) (antibodies are listed in **Table S4**) at 4^•^ C overnight. β-actin served as a loading control. The membranes were then washed in PBST three times (10 minutes each) and incubated with corresponding secondary antibodies conjugated with horseradish peroxidase (HRP) for an hour at room temperature. The membranes were thoroughly washed again with PBST (3 x 15 minutes) to remove the non-specific binding of the secondary antibodies. Signals were captured by the addition of enhanced Chemiluminescence (ECL) reagent (Thermo Scientific, Rockford, IL, USA).

**Generation of the U-118MG brain tumor xenografts.** All animal experiments were reviewed and approved by the Institutional Animal Care and Use Committee (IACUC). U-118MG tumor xenografts were generated as described with slight modifications [1]. Briefly, four to six-week-old mice were used for this study. First, mice were anesthetized by intraperitoneal (i.p.) injection of xylazine and ketamine and transferred to a stereotactic frame (Stoelting Co, IL, USA). U-118MG luciferase transfected cells (1.0 x 10^5 in 3 µl of PBS) filled Hamilton syringe (27 gauge needle) was inserted at 2.25-mm lateral and 1-mm dorsal the bregma to a distance of 3.5-mm and then pulled back 0.5-mm to provide the space for cancer cells. The injection was done at the speed of 1 µl/ 3 min, and the needle was removed after 5 minutes. After 10 days, tumor growth was measured using bioluminescence imaging (BLI) in an IVIS Spectrum (Caliper life sciences; PerkinElmer, MA, USA) by the i.p. injection of BBB permeable luciferase substrate, CycLuc1 (100µl of 5mM CycLuc1). The total photon flux of the mice was analyzed by living Image® software (PerkinElmer, MA, USA). Based on the tumor size, mice were randomized to treatment with (i) vehicle control, (ii) TMZ (25 mg/kg BW, oral gavage), (iii) PCI-24781 (12.5 mg/kg BW, two times per day, oral gavage), iv) vorinostat (100 mg/kg/BW, intraperitoneal), or iv) combination of TMZ + PCI or TMZ + vorinostat for 5 days a week for a month. TMZ + PCI-24781 treated mice were sacrificed after 92 days, and vehicle-treated, and other drug-treated group mice were sacrificed when they were weak.

References

1. Vengoji R, Macha MA, Nimmakayala RK, Rachagani S, Siddiqui JA, Mallya K, Gorantla S, Jain M, Ponnusamy MP, Batra SK *et al*: Afatinib and Temozolomide combination inhibits tumorigenesis by targeting EGFRvIII-cMet signaling in glioblastoma cells. *J Exp Clin Cancer Res* 2019, 38(1):266. 10.1186/s13046-019-1264-2.

2. Elangovan S, Hsieh TC, Wu JM: Growth inhibition of human MDA-mB-231 breast cancer cells by delta-tocotrienol is associated with loss of cyclin D1/CDK4 expression and accompanying changes in the state of phosphorylation of the retinoblastoma tumor suppressor gene product. *Anticancer Res* 2008, 28(5A):2641-2647.

3. Bafna S, Kaur S, Momi N, Batra SK: Pancreatic cancer cells resistance to gemcitabine: the role of MUC4 mucin. *Br J Cancer* 2009, 101(7):1155-1161. 10.1038/sj.bjc.6605285.

4. Chappell WH, Gautam D, Ok ST, Johnson BA, Anacker DC, Moody CA: Homologous Recombination Repair Factors Rad51 and BRCA1 Are Necessary for Productive Replication of Human Papillomavirus 31. *J Virol* 2015, 90(5):2639-2652. 10.1128/JVI.02495-15.

5. Quinn JE, James CR, Stewart GE, Mulligan JM, White P, Chang GK, Mullan PB, Johnston PG, Wilson RH, Harkin DP: BRCA1 mRNA expression levels predict for overall survival in ovarian cancer after chemotherapy. *Clin Cancer Res* 2007, 13(24):7413-7420. 10.1158/1078-0432.CCR-07-1083.

6. Pabla N, Bhatt K, Dong Z: Checkpoint kinase 1 (Chk1)-short is a splice variant and endogenous inhibitor of Chk1 that regulates cell cycle and DNA damage checkpoints. *Proc Natl Acad Sci U S A* 2012, 109(1):197-202. 10.1073/pnas.1104767109.

7. Jin Y, Xu X, Wang X, Kuang H, Osterman M, Feng S, Han D, Wu Y, Li M, Guo H: Increasing sensitivity to DNA damage is a potential driver for human ovarian cancer. *Oncotarget* 2016, 7(31):49710-49721. 10.18632/oncotarget.10436.

8. Pothuraju R, Rachagani S, Krishn SR, Chaudhary S, Nimmakayala RK, Siddiqui JA, Ganguly K, Lakshmanan I, Cox JL, Mallya K *et al*: Molecular implications of MUC5AC-CD44 axis in colorectal cancer progression and chemoresistance. *Mol Cancer* 2020, 19(1):37. 10.1186/s12943-020-01156-y.

9. Nimmakayala RK, Seshacharyulu P, Lakshmanan I, Rachagani S, Chugh S, Karmakar S, Rauth S, Vengoji R, Atri P, Talmon GA *et al*: Cigarette Smoke Induces Stem Cell Features of Pancreatic Cancer Cells via PAF1. *Gastroenterology* 2018, 155(3):892-908 e896. 10.1053/j.gastro.2018.05.041.

**Table S1**

**Drugs common across the four datasets**

| **Drug** | **Mode of action** |
| --- | --- |
| TG-101348 | JAK inhibitor, FLT3 inhibitor, RET tyrosine kinase |
| NCH-51 | HDAC inhibitor |
| Trichostatin –A | HDAC inhibitor |
| Apicidin | HDAC inhibitor |
| Panobinostat | HDAC inhibitor |
| Neratinib | Pan-EGFR inhibitor |
| THM-I-94 (PCI-24781) | HDAC inhibitor |
| ISOX | HDAC inhibitor |
| Belinostat | HDAC inhibitor |
| Vorinostat | HDAC inhibitor |
| Scriptaid | HDAC inhibitor |
| Dacinostat | HDAC inhibitor |

**Table S2**

**Inhibitor selectivity**

| **Compound** | **HDAC** | | | | | | | | | | | **HDAC**  **Family** |
| --- | --- | --- | --- | --- | --- | --- | --- | --- | --- | --- | --- | --- |
|  | **1** | **2** | **3** | **4** | **5** | **6** | **7** | **8** | **9** | **10** | **11** |  |
| Vorinostat |  |  |  |  |  |  |  |  |  |  |  | **++++** |
| Belinostat |  |  |  |  |  |  |  |  |  |  |  | **+++** |
| Panobinostat |  |  |  |  |  |  |  |  |  |  |  | **++++** |
| Trichostatin A  (TSA) |  |  |  |  |  |  |  |  |  |  |  | **++++** |
| ISOX |  |  |  |  |  | **++++** |  |  |  |  |  |  |
| Dacinostat |  |  |  |  |  |  |  |  |  |  |  | **+++** |
| Apicidin |  |  |  |  |  |  |  |  |  |  |  | **√*** |
| Scriptaid |  |  |  |  |  |  |  |  |  |  |  | **√** |
| NCH-51 | **+++** |  |  | **+++** |  | **+++** |  |  |  |  |  |  |
| Abexinostat  (PCI-24781) | **++++** | **+++** | **++++** |  |  | **+++** |  | **++** |  | **+++** |  |  |

"√" compounds which displayed inhibitory effects, but without specific value

**Table S3**

**PCI-24781 inhibits GBM cell viability**

| **Cell line** | **IC25 concentration (µM)** | |
| --- | --- | --- |
|  | **TMZ** | **PCI-24781** |
| U87 | 25 | 0.5 |
| U87vIII | 300 | 0.5 |
| U251 | 35 | 0.625 |
| U251vIII | 125 | 0.625 |
| U118-MG | 500 | 1.25 |

**Table S4**

**List of antibodies used in this study**

| **S. No** | **Antibody name** | **Manufacturer** | **Cat. No** |
| --- | --- | --- | --- |
| 1 | Cleaved caspase 3 | CST | 9664 |
| 2 | Cleaved PARP | CST | 9541 |
| 3 | Phospho-Histone H2A.X(Ser139) | CST | 2577 |
| 4 | β-actin | Sigma | A1978 |
| 5 | RAD51 | CST | 8875 |
| 6 | BRCA1 | Santa Cruz | SC-6954 |
| 7 | CHK1 | Santa Cruz | SC-8408 |
| 8 | Acetylated α tubulin | CST | 5335 |
| 9 | Acetyl histone 3 | CST | 9677 |
| 10 | MGMT | ABclonal | A0693 |
| 11 | Ku70 | Santa Cruz | SC-56129 |
| 12 | HNE | Alpha diagnostic international | HNE11-S |

**Supplementary Table legends:**

**Table S1:** Drugs common across the four datasets are listed.

**Table S2**: Inhibitor selectivity. Table is drawn based on the available information from Selleckchem.com and available literature.

**Table S3:** **PCI-24781 inhibits GBM cell viability**. Drug inhibitory concentration 25 (IC_25_) was determined by MTT assay. MTT-3-(4,5-Dimethyl-2-thiazolyl)-2,5-diphenyl-2H-tetrazolium bromide.

**Table S4:** List of antibodies used in this study.

**Supplementary Figure legends:**

**Figure S1:** **PCI-24781 decreases the viability of MGMT expressing human U-118MG and EGFRvIII, expressing mouse syngeneic GBM cells. (A & B)** Relative cell viability (%) as determined by MTT assay of U-118MG cells treated with the indicated concentrations of PCI -24781 (**A**) and TMZ (**B**) for 48 hours and 72 hours. Viability decreased in mouse syngeneic cell line ((EGFRvIII+, p16^Flox/Flox^, GFAP Cre +).; unpublished data) in a dose-dependent manner with PCI- 24781 (**C**) and not with TMZ (**D**). Data presented as mean +/- SD from experiments done minimally in triplicate.

**Figure S2:** **PCI-24781 + TMZ combination significantly decreases the tumorigenicity of EGFRvIII+, p16^Flox/Flox^, GFAP Cre +** **mouse syngeneic GBM cells.**

Cells were seeded in DMEM complete media. After 12 hours, cells were treated with vehicle control, 300µM of TMZ, 0.5µM of PCI-24781, or TMZ and PCI-24781 for 48 hours, and cultured up to 2 weeks in drug-free DMEM complete media. Colonies were fixed with methanol and stained with crystal violet, then dissolved in 10% acetic acid, and absorbance measured at 595 nm. Results represented as difference in percentage colony formation. (A) Representative images of colony formation assay. (B) Mean percent difference in colony formation in different drug treatment groups from three independent experiments. ANOVA was used to compare the colony formation variable on the natural log scale. Pairwise comparisons are adjusted with Tukey’s method. ‘*’ p ≤ 0.0001; ‘$’ p ≤ 0.001; ‘#’ p < 0.0001 “*” significantly different compared to vehicle control; “$” significantly different compared to TMZ; “#” significantly different compared to PCI-24781.

**Figure S3:** **PCI-24781 shows strong synergistic effects with TMZ in GBM cells.** (**A - B**) U-118MG cells were treated with 250 µM TMZ and denoted concentrations of PCI-24781 for 48 hours, and viable cells were measured by MTT assay. The combination index (CI) was determined using CompuSyn software. (**A**) CI data for non-constant combination TMZ + PCI. (**B**) CI graph reveals the synergy of TMZ and PCI-24781 in U-118MG cells. T- TMZ, P – PCI-24781.

**Figure S4:** **PCI-24781 induces nuclear enlargement in U-118MG cells.** The quantitative measurement of nuclei is represented as relative size compared between control and treatment groups. Student t-test was used to comparisons between control and treatment groups. “*” significantly different compared to vehicle control; “$” significantly different compared to TMZ;

**Figure S5: Genotyping of GEM GBM model.** Total genomic DNA was isolated from mouse tail using Maxwell® Mouse Tail DNA Purification Kit (Promega, Madison, USA) in a Promega Maxwell 16 DNA extraction system (Promega, Madison, USA) as per manufacture’s instruction. Genotyping PCR was done according to animal strain suppliers (NCI Mouse Repository & The Jackson laboratory). Representative genotyping PCR images of (**A**) PTEN, (**B**) EGFRvIII, (**C**) p16, and (**D**) GFAP Cre. WT- wild type; KO- knock out.
