# Supplementary figures and images for: Differential gene expression-based connectivity mapping identified novel drug candidate and improved Temozolomide efficacy for Glioblastoma
Source: J Exp Clin Cancer Res. 2021 Oct 25;40:335. doi: 10.1186/s13046-021-02135-x (PMC8543939; doi:10.1186/s13046-021-02135-x)

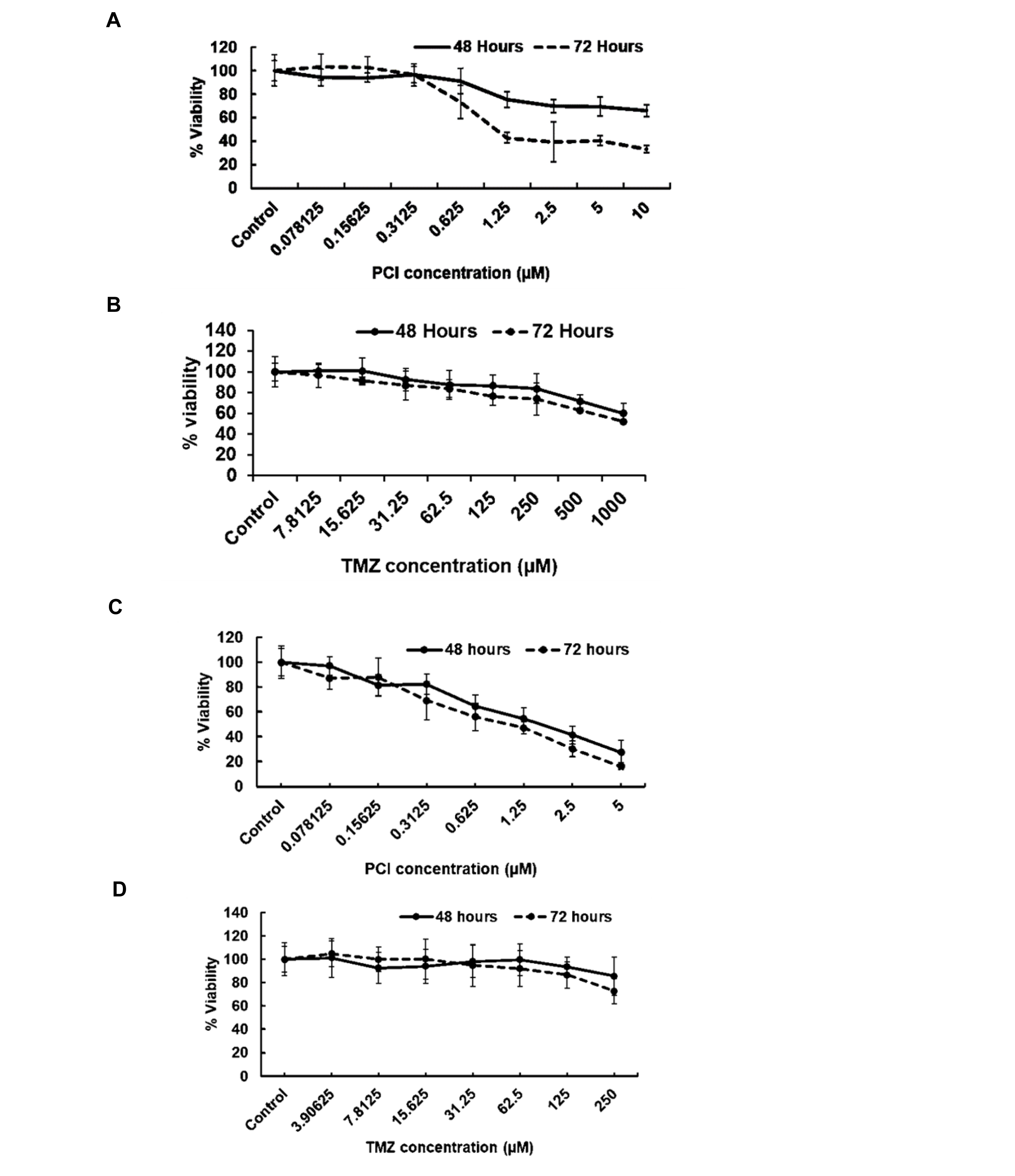

Supplement: Supplementary file 1 — Additional file 1: Supplementary methods. Table S1 Drugs common across the four datasets. Table S2 Inhibitor selectivity. Table S3 PCI-24781 inhibits GBM cell viability. Table S4 List of antibodies used in this study. Figure S1: PCI-24781 decreases the viability of MGMT expressing human U-118MG and EGFRvIII, expressing mouse syngeneic GBM cells. Figure S2: PCI-24781 + TMZ combination significantly decreases the tumorigenicity of EGFRvIII+, p16Flox/Flox, GFAP Cre + mouse syngeneic GBM cells. Figure S3: PCI-24781 shows strong synergistic effects with TMZ in GBM cells. Figure S4: PCI-24781 induces nuclear enlargement in U-118MG cells. Figure S5: Genotyping of GEM GBM model. [file 13046_2021_2135_MOESM1_ESM.zip › FIGURE S1.tif]

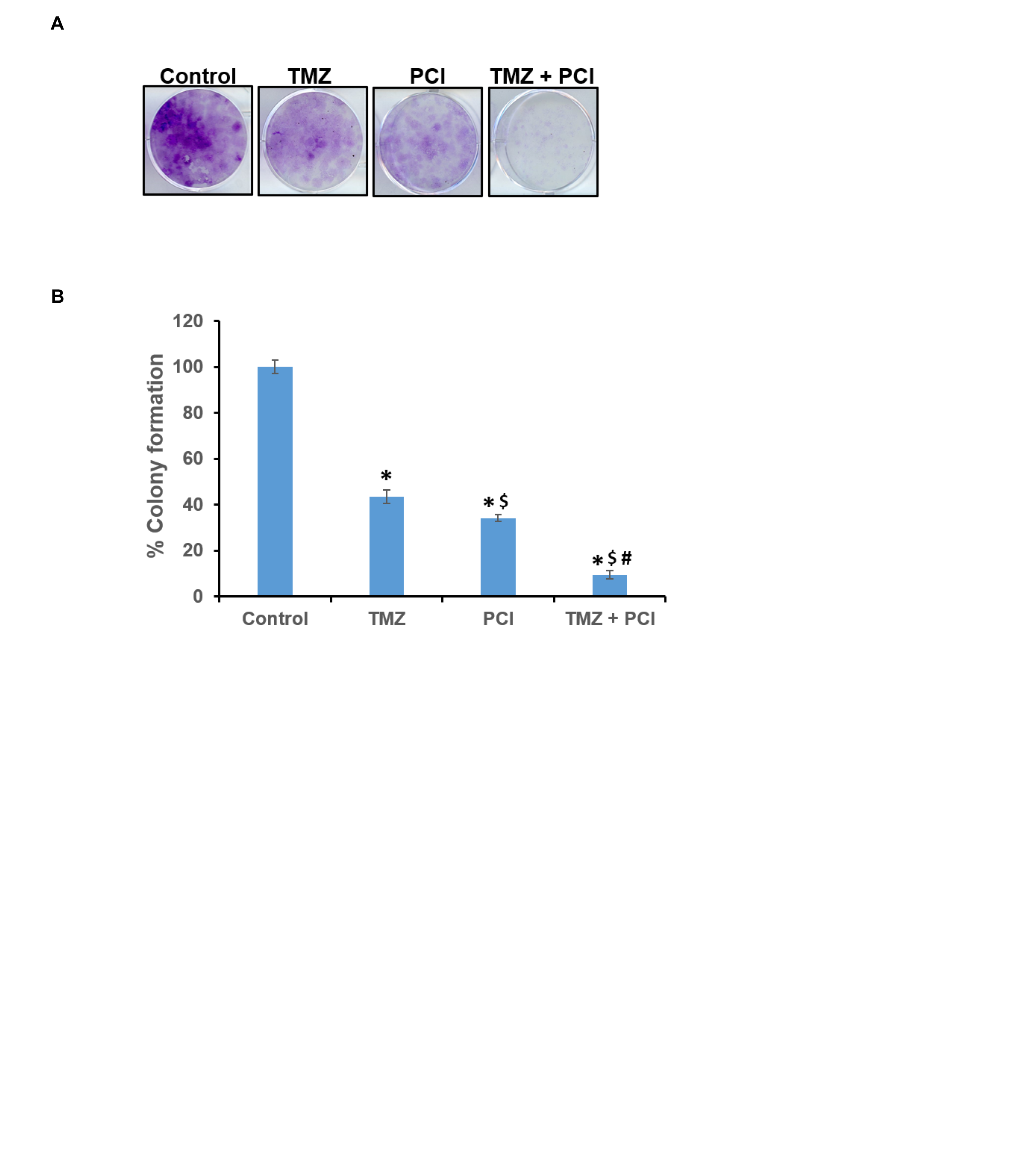

Supplement: Supplementary file 1 — Additional file 1: Supplementary methods. Table S1 Drugs common across the four datasets. Table S2 Inhibitor selectivity. Table S3 PCI-24781 inhibits GBM cell viability. Table S4 List of antibodies used in this study. Figure S1: PCI-24781 decreases the viability of MGMT expressing human U-118MG and EGFRvIII, expressing mouse syngeneic GBM cells. Figure S2: PCI-24781 + TMZ combination significantly decreases the tumorigenicity of EGFRvIII+, p16Flox/Flox, GFAP Cre + mouse syngeneic GBM cells. Figure S3: PCI-24781 shows strong synergistic effects with TMZ in GBM cells. Figure S4: PCI-24781 induces nuclear enlargement in U-118MG cells. Figure S5: Genotyping of GEM GBM model. [file 13046_2021_2135_MOESM1_ESM.zip › FIGURE S2.tif]

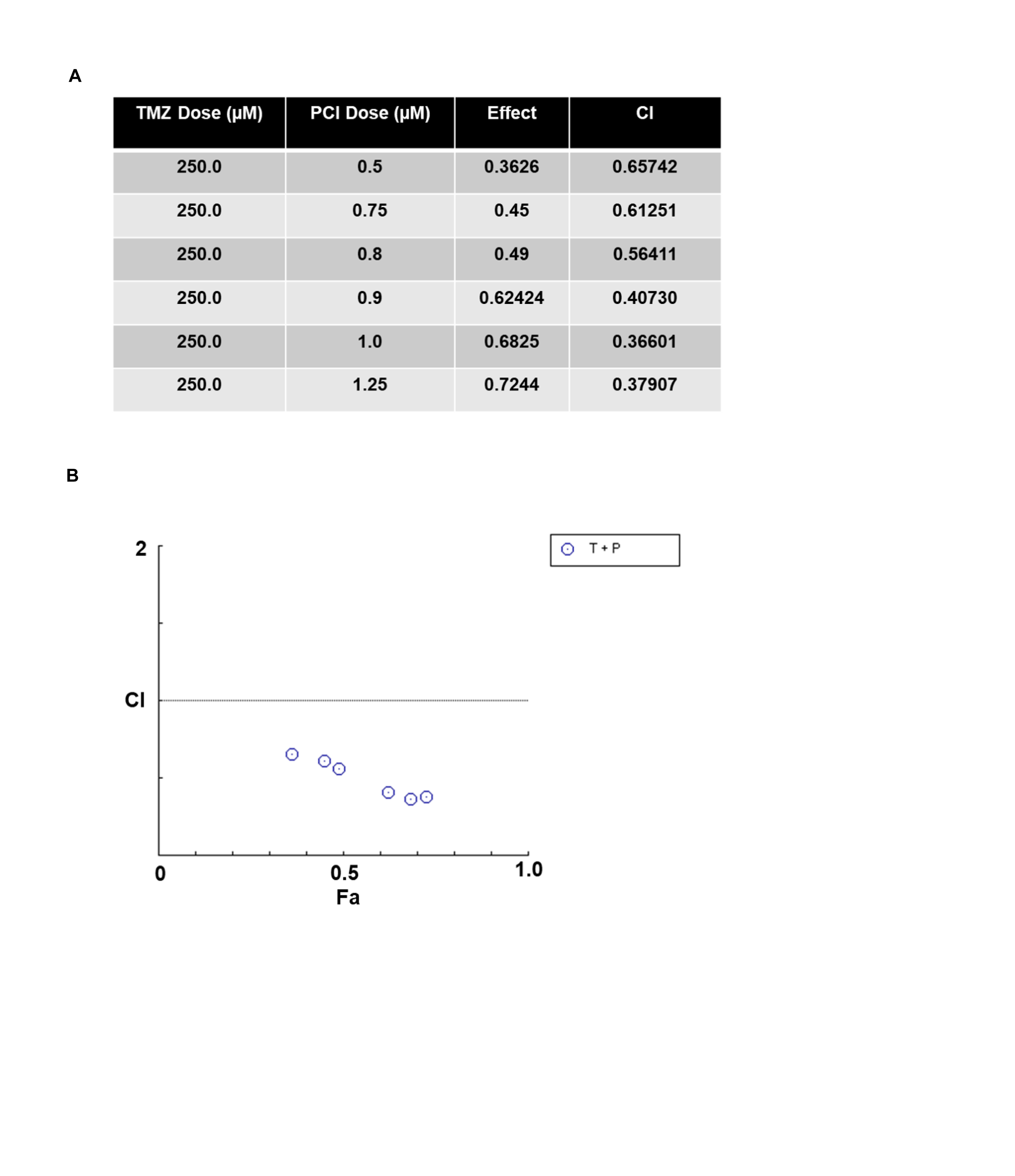

Supplement: Supplementary file 1 — Additional file 1: Supplementary methods. Table S1 Drugs common across the four datasets. Table S2 Inhibitor selectivity. Table S3 PCI-24781 inhibits GBM cell viability. Table S4 List of antibodies used in this study. Figure S1: PCI-24781 decreases the viability of MGMT expressing human U-118MG and EGFRvIII, expressing mouse syngeneic GBM cells. Figure S2: PCI-24781 + TMZ combination significantly decreases the tumorigenicity of EGFRvIII+, p16Flox/Flox, GFAP Cre + mouse syngeneic GBM cells. Figure S3: PCI-24781 shows strong synergistic effects with TMZ in GBM cells. Figure S4: PCI-24781 induces nuclear enlargement in U-118MG cells. Figure S5: Genotyping of GEM GBM model. [file 13046_2021_2135_MOESM1_ESM.zip › FIGURE S3.tif]

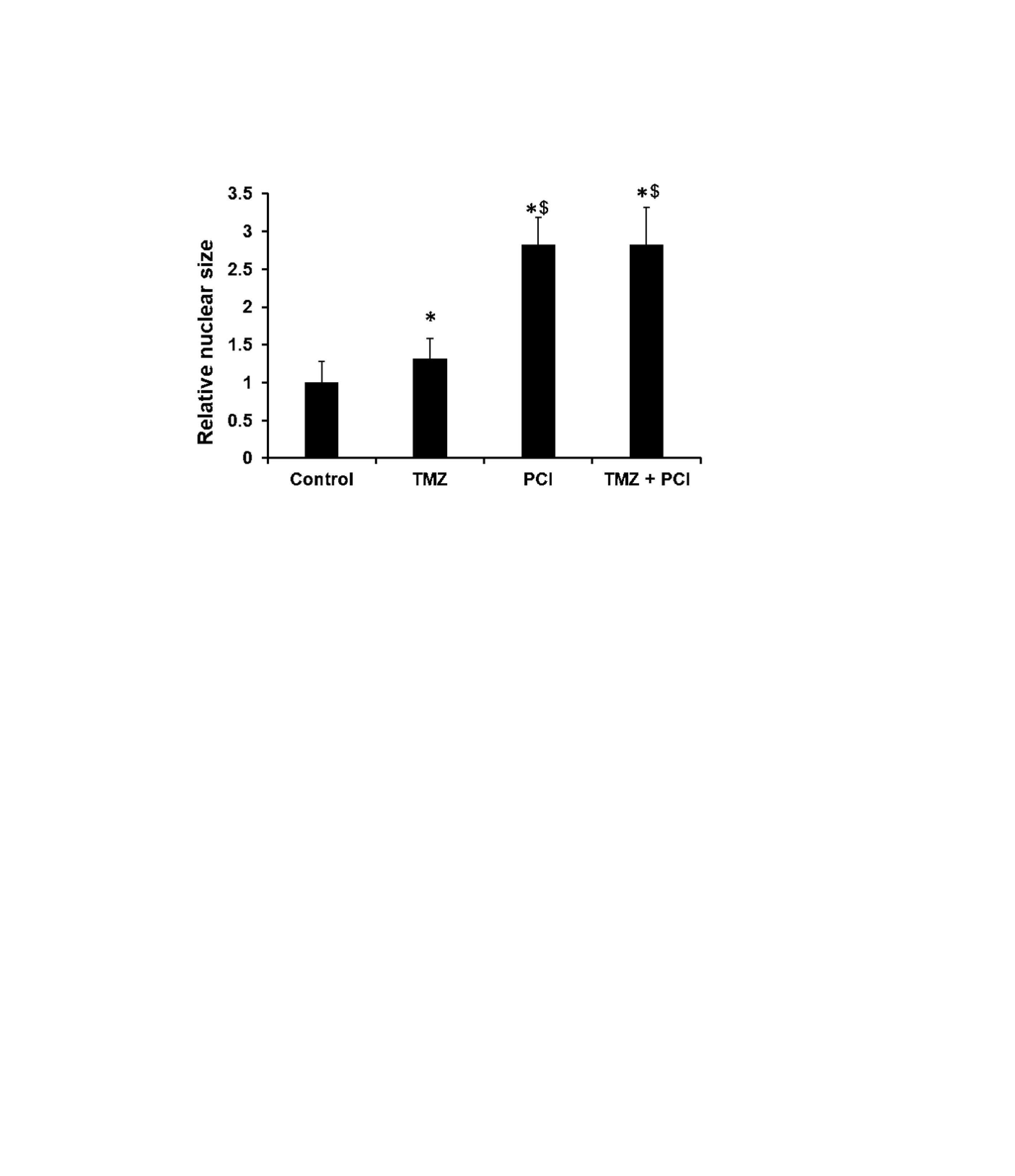

Supplement: Supplementary file 1 — Additional file 1: Supplementary methods. Table S1 Drugs common across the four datasets. Table S2 Inhibitor selectivity. Table S3 PCI-24781 inhibits GBM cell viability. Table S4 List of antibodies used in this study. Figure S1: PCI-24781 decreases the viability of MGMT expressing human U-118MG and EGFRvIII, expressing mouse syngeneic GBM cells. Figure S2: PCI-24781 + TMZ combination significantly decreases the tumorigenicity of EGFRvIII+, p16Flox/Flox, GFAP Cre + mouse syngeneic GBM cells. Figure S3: PCI-24781 shows strong synergistic effects with TMZ in GBM cells. Figure S4: PCI-24781 induces nuclear enlargement in U-118MG cells. Figure S5: Genotyping of GEM GBM model. [file 13046_2021_2135_MOESM1_ESM.zip › FIGURE S4.tif]

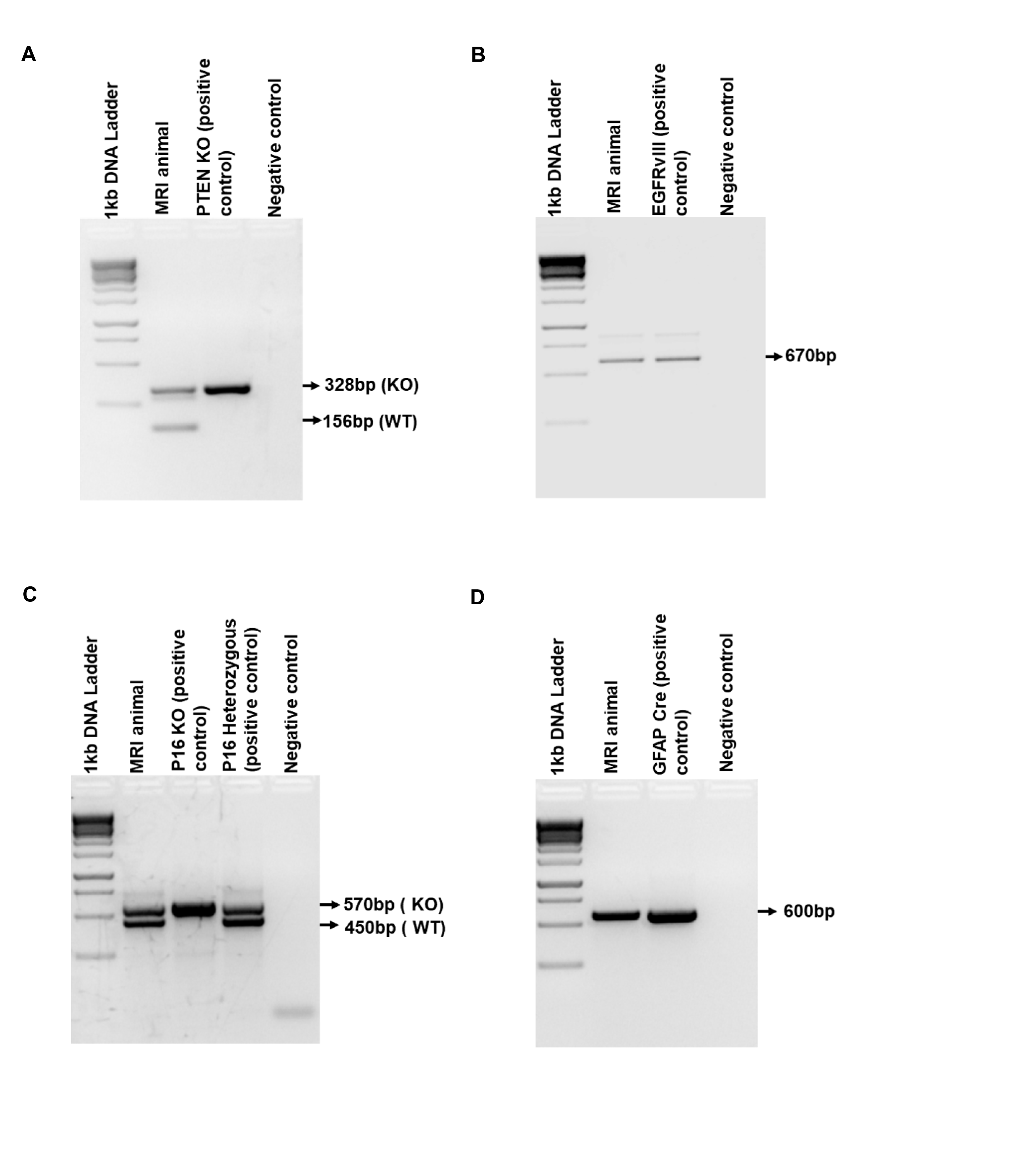

Supplement: Supplementary file 1 — Additional file 1: Supplementary methods. Table S1 Drugs common across the four datasets. Table S2 Inhibitor selectivity. Table S3 PCI-24781 inhibits GBM cell viability. Table S4 List of antibodies used in this study. Figure S1: PCI-24781 decreases the viability of MGMT expressing human U-118MG and EGFRvIII, expressing mouse syngeneic GBM cells. Figure S2: PCI-24781 + TMZ combination significantly decreases the tumorigenicity of EGFRvIII+, p16Flox/Flox, GFAP Cre + mouse syngeneic GBM cells. Figure S3: PCI-24781 shows strong synergistic effects with TMZ in GBM cells. Figure S4: PCI-24781 induces nuclear enlargement in U-118MG cells. Figure S5: Genotyping of GEM GBM model. [file 13046_2021_2135_MOESM1_ESM.zip › FIGURE S5.tif]
